# jClustering, an Open Framework for the Development of 4D Clustering Algorithms

**DOI:** 10.1371/journal.pone.0070797

**Published:** 2013-08-22

**Authors:** José María Mateos-Pérez, Carmen García-Villalba, Javier Pascau, Manuel Desco, Juan J. Vaquero

**Affiliations:** 1 Instituto de Investigación Sanitaria Gregorio Marañón, Madrid, Spain; 2 Centro de Investigación Biomédica en Red de Salud Mental (CIBERSAM), Madrid, Spain; 3 Departamento de Bioingeniería e Ingeniería Aeroespacial, Universidad Carlos III de Madrid, Madrid, Spain; UGent/VIB, Belgium

## Abstract

We present *jClustering*, an open framework for the design of clustering algorithms in dynamic medical imaging. We developed this tool because of the difficulty involved in manually segmenting dynamic PET images and the lack of availability of source code for published segmentation algorithms. Providing an easily extensible open tool encourages publication of source code to facilitate the process of comparing algorithms and provide interested third parties with the opportunity to review code. The internal structure of the framework allows an external developer to implement new algorithms easily and quickly, focusing only on the particulars of the method being implemented and not on image data handling and preprocessing. This tool has been coded in Java and is presented as an ImageJ plugin in order to take advantage of all the functionalities offered by this imaging analysis platform. Both binary packages and source code have been published, the latter under a free software license (GNU General Public License) to allow modification if necessary.

## Introduction

Dynamic nuclear imaging studies have become a common diagnostic technique in medicine, as they provide quantitative and functional information on several tissues thanks to the use of radiolabeled tracers with different *in vivo* behaviors [1]–[6]. In order to obtain accurate kinetic parameters for compartmental models [7], it is first necessary to generate precise time-activity curves (TACs) both for the tissues being studied and for input functions, such as the TACs of the myocardium and ventricles in the case of a cardiac study. These curves can be obtained directly from the image by manually drawing regions of interest (ROIs), although this is a slow, time-consuming, subjective process [8]–[10]. In order to avoid these problems, many automatic or semiautomatic segmentation algorithms have been developed over the years. These algorithms group together regions of the image with similar kinetics in order to obtain mean activity curves and thus improve the signal-to-noise ratio. Examples of these algorithms include principal component analysis (PCA) [11], *k-means* clustering [9], factor analysis [12]–[14], hierarchical clustering [15], [16], leader-follower clustering [17], segmentation based on TAC similarity metrics [18], multiphase level set methods [19] and independent component analysis (ICA) [20], [21].

One of the problems affecting many algorithms is unavailability of source code [22], not even in binary package form. Consequently, interested researchers, who may not have a technical background, are forced to re-implement the algorithms in order to use them or perform comparisons with their own methods. Algorithm reimplementation requires programming knowledge and is open to errors.

As a preliminary test, the source code for 11 previously published articles on new dynamic positron emission tomography (PET) segmentation algorithms (all published after 2002) was requested by e-mail, and all responses were gathered over a one-month period. Four error messages were received, because the e-mail address was no longer valid. One respondent stated that the algorithm was patented and therefore no source code could be provided, one claimed that the code was already obsolete, one reported that the code had been developed by another person and refused, and three e-mails went unanswered or were answered once with no follow-up. One author sent the requested code.

This paper presents *jClustering*, an open source tool and framework developed to facilitate implementation of segmentation algorithms for dynamic molecular imaging, but that can be potentially used for any dynamic medical imaging modality, such as dynamic contrast-enhanced magnetic resonance studies. In order to accomplish this purpose, the tool was written in Java, a programming language that does not require any kind of use fee and has an internal structure that lets the developer or researcher concentrate on the specifics of the algorithm. Furthermore, it is published under GNU GPL, a free software license, to allow code reviews and modification by interested third parties.

## Materials and Methods

### Programming languages and design considerations

As *jClustering* was designed to simplify the implementation of new segmentation algorithms in dynamic nuclear medicine studies, image handling (eg, loading, saving, displaying) was separated as much as possible from segmentation. Therefore, it was decided that this tool would be developed as an ImageJ plugin [23]. ImageJ is an imaging processing platform developed by the National Institutes of Health (Bethesda, Maryland, USA) with a very active community of users and developers and many different plugins and macros developed by this community [24]. It provides an open and stable application programming interface (API) that performs the background tasks and allows easy and reliable 4D (3D plus time) image manipulation.

The tool presented here was developed using Java (Oracle Corporation, Santa Clara, California, USA), as ImageJ is written in this programming language. Developing with Java is free and therefore fits with the objectives of the project.

### Processing workflow overview

The process of generating cluster images by temporal similarity involves the analysis of all the TACs in order to group them into different classes, each with a mean activity curve, according to a specific algorithm. These classes are then said to define different regions in the subject according to variations in their kinetics.

**Figure 1:**
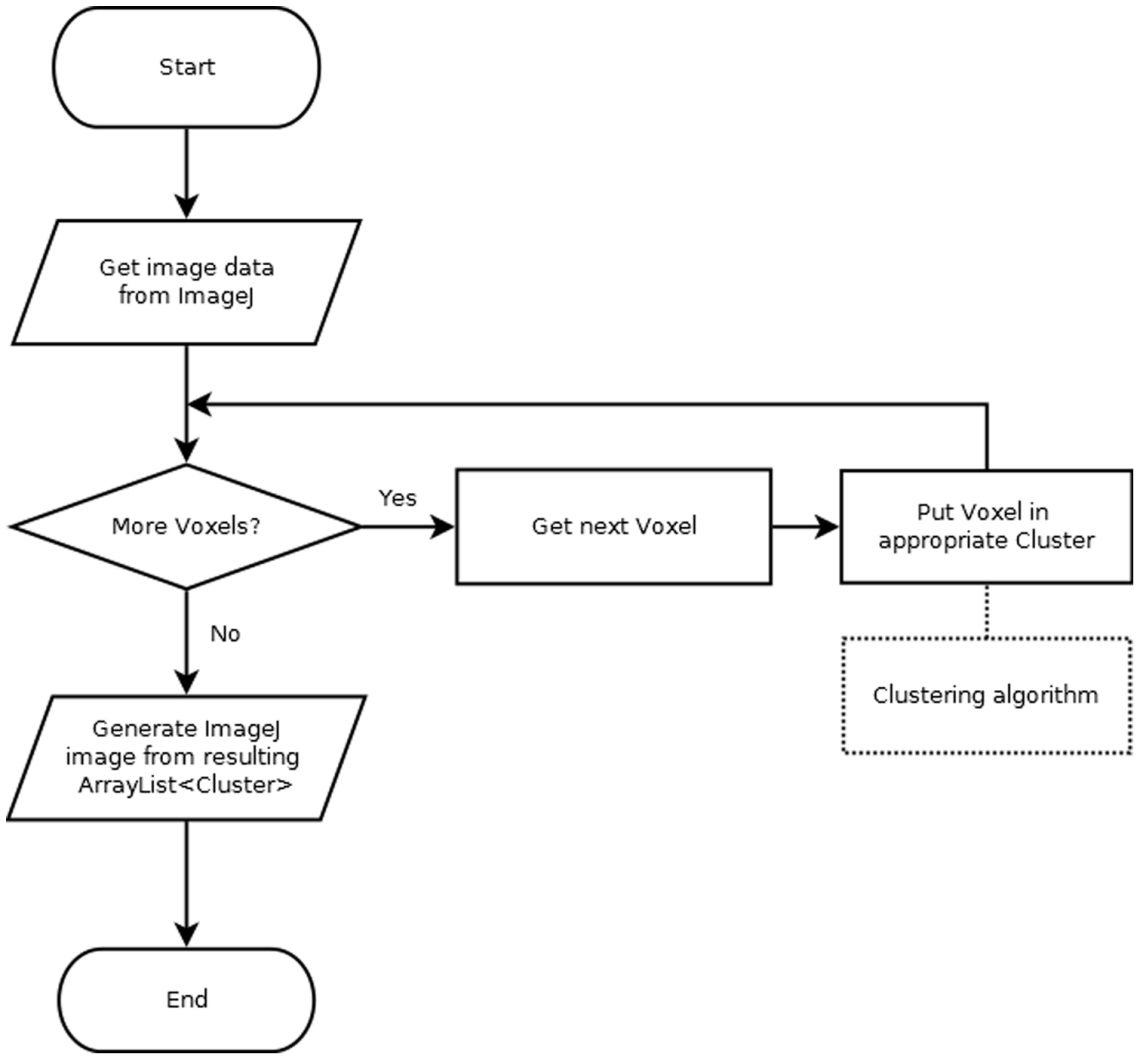
Basic flow diagram. Flow diagram of the basic steps necessary to perform a clustering operation. In iterative algorithms, several loops of the voxel assignation stage can be performed until convergence is reached.

The workflow implemented was kept as simple as possible and is depicted in Figure 1. In short, each individual voxel TAC is passed to the *ClusteringTechnique* module, which can re-use a *ClusteringMetric* if the metric of a particular algorithm has already been used. This *ClusteringTechnique* module groups together objects of the class *Voxel* (which contains TAC data and spatial information) using the *Cluster* class and adds all the formed *Cluster* objects to a native *ArrayList* object. Then, the final *ArrayList* object is automatically converted to an *ImagePlus* object for cluster visualization, since it is a native ImageJ image object. In order to present the clusters comprehensively, a pseudo-dynamic image containing *n+1* frames is used, with *n* being the total number of clusters formed. The *n^th^* frame contains the visual information for the *n^th^* cluster, and the last frame contains a simultaneous composition of all the clusters for better spatial reference. This simplified workflow will be expanded in the following section as the relevant classes are discussed. The full public API can be found in File S1.

### Relevant implemented classes and methods

#### ImagePlusHyp

The clustering algorithm reads the 3D image temporal sequence as a set of individual TACs, each of which corresponds to a single voxel.

ImageJ stores 4D images, called HyperStacks, as 3D images in which the slice number is proportional to the number of slices and frames combined (e.g., a HyperStack with 20 slices and 20 frames will contain 400 slices); therefore, obtaining the temporal TAC values for a given voxel involves inspecting the slices in the correct order. To simplify this procedure, a wrapping class (*ImagePlusHyp*) was created. This class serves as a proxy interface for the native ImageJ classes *ImagePlus* and *ImageStack*, thus enabling them to be handled efficiently for this TAC extraction task; the developer only needs to provide the coordinates for the desired voxel using the *getTac(int x, int y, int slice)* method, and the corresponding TAC will be returned. Furthermore, this class provides an *ImagePlusHypIterator* object that implements an *Iterator* which returns, one by one, all the voxels within the image for convenient use inside loops. The voxel information is contained in a *Voxel* class that stores a reference to the TAC data as a *double[]* and the *x*, *y*, and *slice* coordinates (the *slice* coordinate can be thought of as a 1-based *z* coordinate, or *z+1*), in case the spatial information is needed.

#### Cluster

The *Cluster* class represents a grouping of voxels defined by a mean TAC known as a *centroid*. A *Cluster* may work in two different ways: either an invariant centroid is generates upon creation of the cluster and serves as a fixed reference or it is modified as new voxels are added to the cluster. This behavior is controlled by the constructor used: *Cluster()*, *Cluster(double [] centroid, int x, int y, int slice)*, and *Centroid(Voxel v)* create a *Cluster* object that will modify the centroid with each new addition. Such an approach is valid, given that a *Cluster* that is created from a single voxel is not using the centroid from a previous *Cluster* and may therefore be subject to change. *Cluster(double [] centroid)*, on the other hand, creates a *Cluster* with an immutable reference centroid and computes a mean cluster TAC with each addition.

#### ClusteringTechnique

*ClusteringTechnique* is an abstract class containing methods that must be implemented by extending classes in order to perform the actual clustering. It is also the main class, and often the only one that an external developer should extend when implementing a new clustering algorithm.

Internally, the tool creates an instance of the chosen extending class and initializes certain internal values so that the object is in a consistent state, including a reference to a *ClusteringMetric* (if needed), a reference to an initialized but empty *ArrayList<Cluster>* that will contain the *Cluster* objects generated, and a reference to the image data in the form of an *ImagePlusHyp*.

The only method that must be implemented is *process()*, which must fill in the *ArrayList<Cluster>* object with the appropriate *Cluster* objects. Should the algorithm require user input, the *makeConfig()* method, which returns a *JPanel*, must also be implemented, although user input is completely optional.

Although the *Cluster* objects will be automatically shown on screen with the correct formatting, the developer may also show additional images using the ImageJ native methods at this point, if necessary. Also, a *String[]* object can be filled with additional information; if present, this information will be saved along with the TAC data in the same directory.

#### ClusteringMetric

In order to promote re-use of code, the *ClusteringMetric* abstract class was implemented. Some clustering algorithms, such as k-means, group voxels together according to a specific distance, which may be the Euclidean distance, the correlation or covariance scores between two given TACs, or more elaborate metrics such as the Mahalanobis distance. Algorithms such as k-means can benefit from sharing code in the form of a *ClusteringMetric*, which computes the distance between two given TACs and only needs to be implemented once.

The *ClusteringMetric* abstract class has only one method that must be extended, namely, *distance(double [] a, double [] b)*, which computes the distance between these two arrays. As in the previous class, if a configuration dialog is needed, the developer can implement the *makeConfig()* method.

As some metrics (e.g., the Mahalanobis distance) may need to process initial data (in this case, the covariance matrix for the image), an *init()* method is provided; for this purpose, the *ClusteringMetric* objects also contain a reference to enable access to all the image data. This method is called once by the *ClusteringTechnique* before any call to the *distance()* method and can be used to initialize the necessary variables.

**Figure 2:**
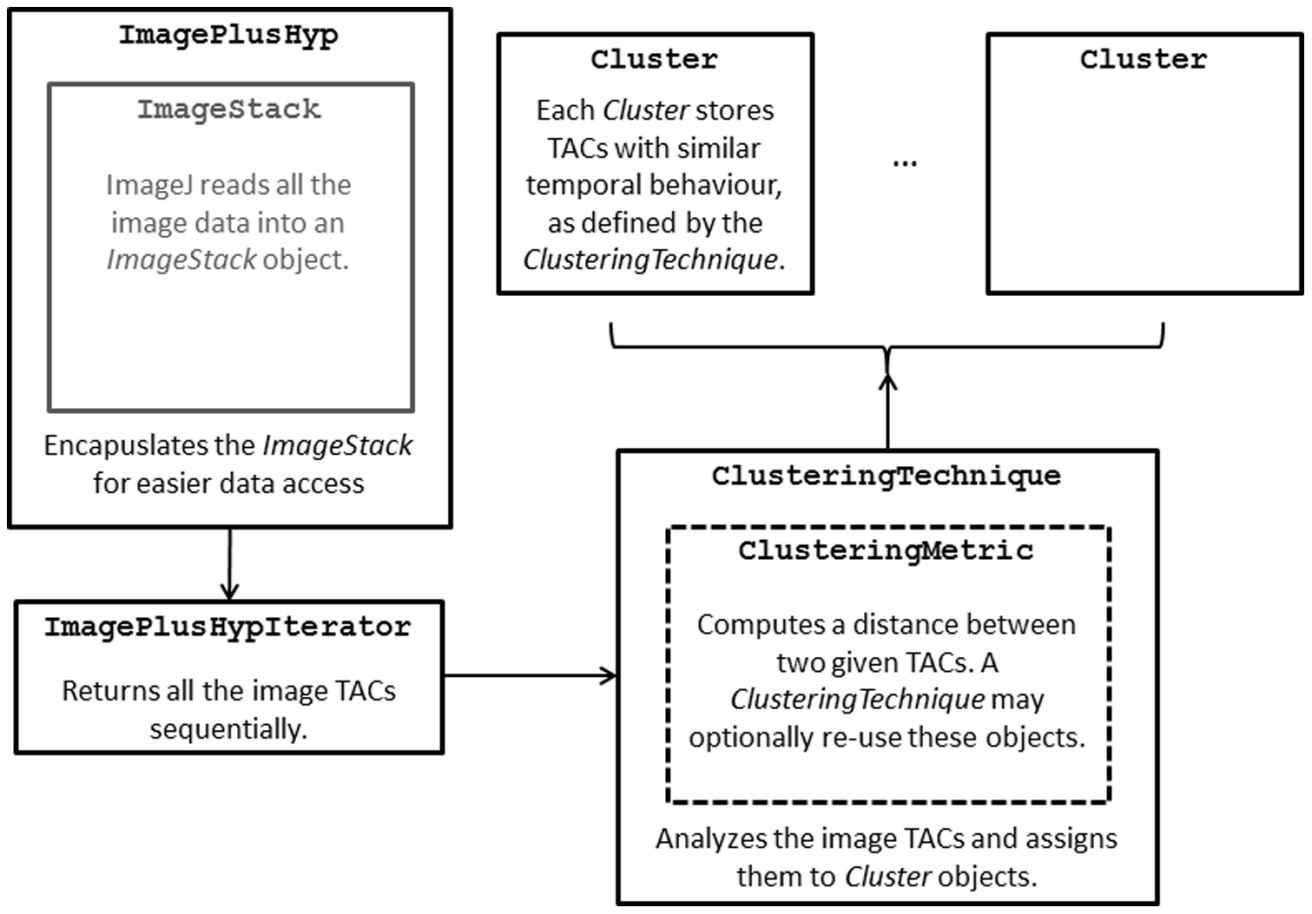
Diagram of the main classes. Only the most relevant classes implemented are included in the diagram. Helper classes (eg, for GUI building or mathematical libraries) are not shown.

Figure 2 shows a diagram of the relationships between these classes.

### Algorithms implemented

In order to provide an example of the capabilities of the framework, several clustering algorithms and metrics were implemented, as follows: *k-means* [25], *k-means++* [26], *leader-follower* [17], PCA [11], singular value decomposition (SVD) [25] and ICA [20]. The metrics implemented, which can currently be used by the *k-means* algorithm, are the Pearson correlation score, p-norm (eg, p = 1 for Manhattan distance and p = 2 for Euclidean distance), and Mahalanobis distance. The Pearson correlation distance is computed as
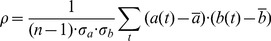
(1)where *a(t)* and *b(t)* are the two TACs, 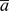
and 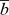
are the mean values for those TACs and 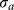 and 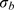
are the standard deviations. In order to turn the correlation into a distance, the method returns 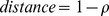.

The generalized p-norm metric is defined as
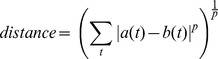
(2)


This metric has a configuration panel that allows the user to set the value for the *p* parameter (defaults to 2.0, Euclidean distance).

Finally, the Mahalanobis distance is defined as
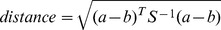
(3)where *S* is the covariance matrix for the image; to speed up computation, this matrix is computed just once, using the *init()* method.

### Graphical User Interface (GUI)

A simple GUI was built. It contains three separate tabs: the main configuration tab, the technique configuration tab, and the metric configuration tab (the last two tabs may be empty if no configuration is needed). The main configuration tab makes it possible to select a directory in which to store a text file. This text file stores the values of the TACs for every cluster so that they can be used as input in subsequent analyses. *jClustering* can store this text file in comma- or tab-separated values or in PMOD (PMOD Technologies Ltd., Zurich, Switzerland) format.

ImageJ does not extract each frame duration from the image metadata. This problem was solved by complementing the main interface with a selection button to provide a file containing the frame start and end times in a tab- or space-separated text file with two columns and as many rows as frames. The resulting temporal data, which are essential for kinetic analysis, are stored in the first two columns of the resulting text file that contains the different TACs and can then be entered into a kinetic analysis program such as PMOD.

### Input image formats

One of the advantages of developing tools for *ImageJ* is that the package manages image I/O. *jClustering* is able to deal with any image format that *ImageJ* can open, provided the contents are a dynamic image, either 2D + time or 3D + time; upon opening a file, the image data is internally assigned to an *ImagePlus* object that is directly handled by *jClustering* classes. If the image is static, a warning is shown. The test images used in this paper are stored in either DICOM or Analyze format; in other tests, raw images have been converted to an *ImageJ* HyperStack and processed.

### Class autodetection

New *ClusteringTechnique* and *ClusteringMetric* child classes are automatically detected if they belong to their corresponding packages and are stored in the right directories (*jclustering/techniques* and *jclustering/metrics*, respectively) and the necessary GUI elements are updated accordingly. External developers are thus freed from the added burden of having to modify the core *jClustering* files to add their own classes.

### Licensing

To ensure that third parties are able not only to extend but also to modify and adapt this tool, a free license is the best option. In this case, the source code is licensed using a GNU General Public License (GPL).

Even though development started privately, once a stable release could be provided, all the code was copied to a public git repository available at [27] and all subsequent development was public.

### Installation

jClustering installation is straightforward, considering it has been coded as a plugin for the ImageJ platform. Users need to download the latest jClustering_.jar file from the download site [28] and copy it to their *plugins/*directory in their local ImageJ installation. Apache Commons Math and FastICA libraries are also needed; they must be copied in the *plugins/jars/*directory in their local ImageJ installation. A link to these libraries is provided in the main jClustering page github page.

Once these files have been copied, jClustering can be run from the main ImageJ menu under *Plugins > Clustering*.

The source code is provided as a Maven repository, which allows developers to easily create their own projects and compile *jClustering* into a.jar file.

## Results

**Figure 3:**
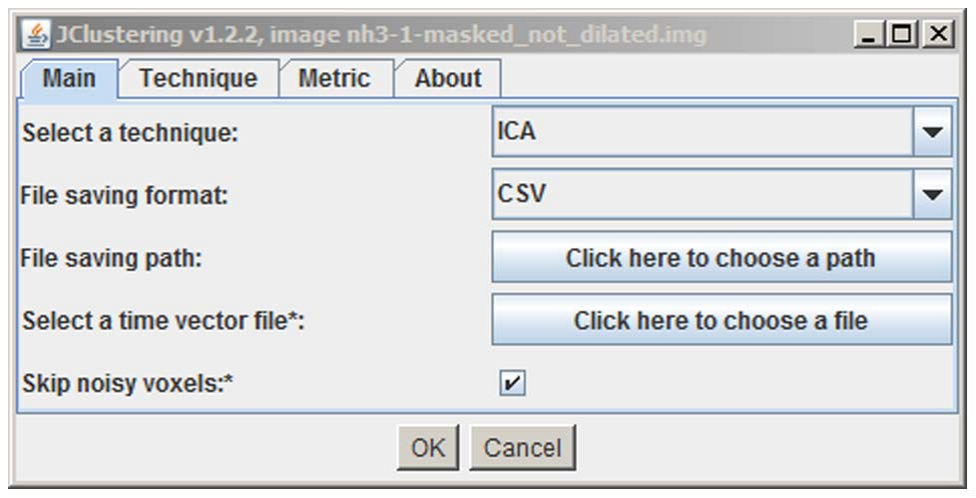
Main program window screenshot. This window allows the user to select the *ClusteringTechnique* and set several options for data output. The top tabs can be used if the *ClusteringTechnique* (and its *ClusteringMetric*, where available) requires input for configuration. As the image is loaded into the plugin upon execution, the original image title is also shown to stress the fact that all the clustering operations will be applied to that image.

The main program window is shown in Figure 3. The different configuration panels are accessible using the top tabs, thus enabling all the necessary options to be presented in a single window.

**Figure 4:**
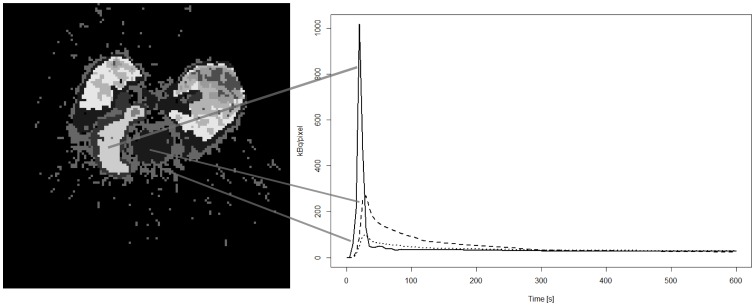
*k-means++* result example. Clustering results for a ^13^NH_3_ pig study using a *k-means++* algorithm (*k* = 10). The myocardium, the right ventricle, the left ventricle, and the lungs are clearly delineated. The activity curves for some relevant regions (right ventricle, left ventricle, and myocardium; right panel) are plotted from the text file stored by *jClustering* after segmentation. The left image shows the last frame of the pseudo-dynamic structure generated to display the results. The value of each voxel in this image contains the cluster number that contains said voxel. Each frame before the last one displays only the voxels that belong to that cluster number.

**Figure 5:**
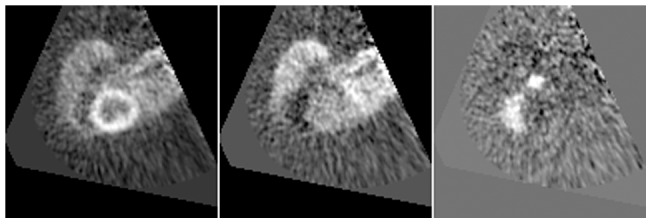
PCA result example showing additional images. Three principal components resulting from applying PCA to the same study as the one used to generate Figure 4. They have been chosen to represent the myocardium (left), blood pool (center) and right ventricle (right). These images are shown during the *process()* method execution, prior to displaying the final clusters.

**Figure 6:**
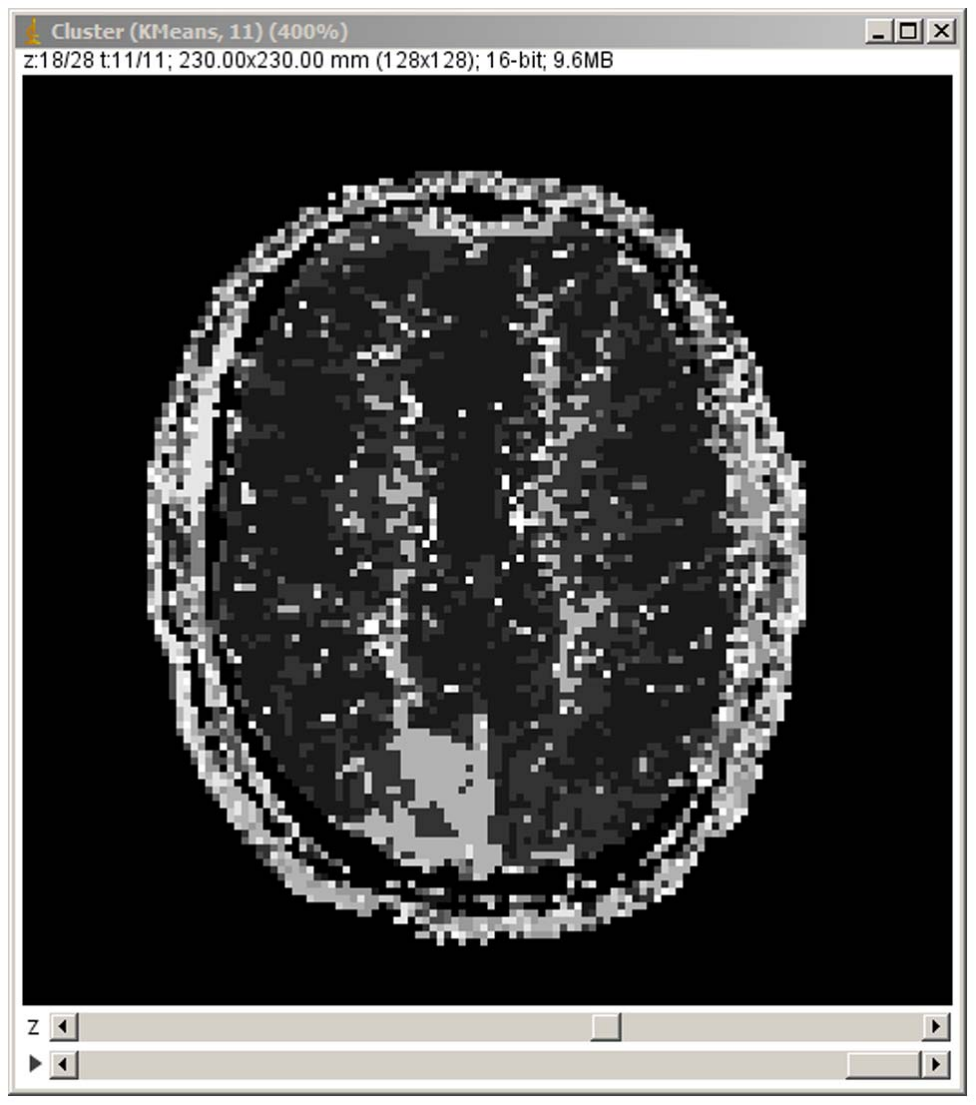
Example of the tool applied to a dynamic magnetic resonance study. Clustering results for a dynamic MRI study using gadolinium as a contrast agent in a human subject. A lesion can be seen clearly at the bottom of the image. In this case, the full image screen is shown to highlight the ImageJ frame and the dynamic image controls that allow the user to switch between the different classes or, as in this case, a last frame containing all the classes, each with a different gray level.

Figure 4 shows a segmentation of a dynamic PET study using a *k-means++* algorithm (*k* = 10) with Euclidean distance as a metric. The image dimensions are 128×128×47, 25 frames, and the total time used in the segmentation is 20.15 seconds. Several principal components from a PCA of this image are shown in Figure 5; a total of 25 principal components were computed in 14.20 seconds. Figure 6 shows a simple segmentation of a dynamic human MRI study with gadolinium as a contrast agent using a grayscale LUT. The image dimensions are 128×128×28, 40 frames, and the total processing time is 10.47 seconds.

## Discussion

Although several papers have been published on automatic or semiautomatic dynamic imaging segmentation, very few include or even make available the source code of the algorithms developed. It can be difficult or even impossible to obtain the source code from the authors, as the contact address is no longer valid, the original code has been lost, or the author refuses to deliver it. Obviating the need to trace an author would leave the researcher free to focus on the solution to the problem and not on avoidable distractions. Occasionally, the source code can be obtained, only to discover that it has been implemented in a programming language that requires a fee for use. The development and publishing of an open platform that addresses this particular drawback makes sense not only from the point of view of offering a standard tool for free development, but also because it encourages code sharing and publication, which creates numerous advantages [22], [29], including the possibility of receiving code reviews from third parties that can disclose previously undetected bugs.

Furthermore, publication of the source code for a new algorithm would help researchers to compare methods without having to re-implement each one using hard copy, which is a slow and error-prone process that could require further programming expertise. It would be better if algorithms could be executed in a common segmentation platform such as the one presented here.

*jClustering* addresses these issues by providing a free and open clustering framework for effortless implementation of new clustering algorithms (see File S2 for a simple example). As image-handling is delegated to ImageJ, new functionalities can be implemented using the remaining structure.

*jClustering* works in the Windows, Linux, and Macintosh operating systems. It does not use machine-dependent code or libraries and runs on the same platforms as ImageJ.

In this paper, *jClustering* is presented using dynamic studies in the context of nuclear medicine. However, Figure 5 (a successfully segmented dynamic perfusion brain study using magnetic resonance imaging) illustrates how this tool can be used with any kind of temporal image sequence.

*jClustering* is subject to a series of limitations. For instance, it cannot perform *fuzzy* clustering, in which every voxel is assigned a probability of belonging to a given cluster. All the segmentations performed with the current structure and class hierarchy associate a voxel with a region in a deterministic way, although it would be possible to implement the necessary changes to allow *fuzzy* clustering to work within this framework. This first approach allows some of the most common clustering algorithms, such as k-means, to be implemented. This drawback is in part mitigated with the possibility of generating additional information, both in image and text form, during the clustering operation (see Figure 5, for example).

Furthermore, *jClustering* cannot obtain the temporal information from the image metadata, which is a fundamental parameter for kinetic analysis. These data must be extracted from the image header, if present, by the user and stored in a text file.

*jClustering* works for ImageJ versions posterior to 1.46r, although it is not ready for the new ImageJ2 branch, which is expected to finish beta testing in June 2014. We decided to use the regular ImageJ distribution, which is currently the most widely used, has a stable application programming interface (API), and will be maintained for years to come.

The current version (1.2.2 at the time of writing) provides a stable API and already contains implementations of *k-means*, *k-means++*, *leader-follower*, ICA, PCA and SVD applied to image segmentation, although more methods should and will be added; hence the development of *jClustering*, a common platform for processing of clusters in dynamic medical imaging.

## Conclusion

*jClustering* is an open framework for the implementation of dynamic imaging segmentation algorithms. It uses *ImageJ* capabilities to open, save, and display images, leaving the developer with the task of implementing new algorithms. Its source code has been made public under a free software license (GNU GPL) and is available, along with documentation and a link to binary releases, at [27].

## Supporting Information

File S1 **Public API for jClustering version 1.2.2.**
(ZIP)

File S2 **Example of simple** ***ClusteringTechnique*** **class.**
(DOCX)
