# Supplementary material for: jClustering, an Open Framework for the Development of 4D Clustering Algorithms
Source: PLoS One. 2013 Aug 22;8(8):e70797. doi: 10.1371/journal.pone.0070797 (PMC3750055; doi:10.1371/journal.pone.0070797)
Supplement: File S1 — Public API for jClustering version 1.2.2. (ZIP) [file pone.0070797.s001.zip › allclasses-frame.html]

All Classes


# All Classes

- Cluster
- ClusteringMetric
- ClusteringTechnique
- Constants
- Correlation
- FileSaver
- GUIUtils
- ICA
- ImagePlusHyp
- ImagePlusHypIterator
- JClustering\_
- KMeans
- LeaderFollower
- Mahalanobis
- MathUtils
- PCA
- PNorm
- RMSD
- SampleTechnique
- SVD
- TimeVectorReader
- Utils
- Voxel
