# Supplementary material for: jClustering, an Open Framework for the Development of 4D Clustering Algorithms
Source: PLoS One. 2013 Aug 22;8(8):e70797. doi: 10.1371/journal.pone.0070797 (PMC3750055; doi:10.1371/journal.pone.0070797)
Supplement: File S1 — Public API for jClustering version 1.2.2. (ZIP) [file pone.0070797.s001.zip › constant-values.html]

Constant Field Values


JavaScript is disabled on your browser.


- Overview
- Package
- Class
- Use
- Tree
- Deprecated
- Index
- Help

- Prev
- Next

- Frames
- No Frames

- All Classes

# Constant Field Values

## Contents

- jclustering.\*

## jclustering.\*

- jclustering.Constants

  | Modifier and Type | Constant Field | Value |
  |  |  |  |
  | --- | --- | --- |
  | `public static final java.lang.String` | `NO_METRIC` | `"No metric selected or no configuration needed"` |
  | `public static final java.lang.String` | `NO_TECHNIQUE` | `"No clustering technique selected or no configuration needed"` |
  | `public static final java.lang.String` | `PACKAGE_NAME` | `"jclustering"` |
  | `public static final java.lang.String` | `VERSION` | `"v1.2.2"` |

- Overview
- Package
- Class
- Use
- Tree
- Deprecated
- Index
- Help

- Prev
- Next

- Frames
- No Frames

- All Classes
