# Supplementary material for: jClustering, an Open Framework for the Development of 4D Clustering Algorithms
Source: PLoS One. 2013 Aug 22;8(8):e70797. doi: 10.1371/journal.pone.0070797 (PMC3750055; doi:10.1371/journal.pone.0070797)
Supplement: File S1 — Public API for jClustering version 1.2.2. (ZIP) [file pone.0070797.s001.zip › index-files/index-1.html]

A-Index


JavaScript is disabled on your browser.


- Overview
- Package
- Class
- Use
- Tree
- Deprecated
- Index
- Help

- Prev Letter
- Next Letter

- Frames
- No Frames

- All Classes

A C D E F G H I J K L M N P R S T U V X Y 


## A

actionPerformed(ActionEvent) - Method in class jclustering.JClustering\_


actionPerformed(ActionEvent) - Method in class jclustering.metrics.ClusteringMetric


add(double[], int, int, int) - Method in class jclustering.Cluster
:   Adds given data to cluster.

add(Voxel) - Method in class jclustering.Cluster
:   Provides a shortcut to the `add(double[], int, int, int)`
    method using a `Voxel`.

addCluster(double[]) - Method in class jclustering.techniques.ClusteringTechnique
:   Creates a new cluster with the `double [] tac` as the centroid,
    adds it to the cluster ArrayList and returns it.

addMetricsToJPanel(JPanel) - Method in class jclustering.techniques.ClusteringTechnique
:   Adds a `JComboBox` element containing all the available metrics to the
    given panel.

addTACtoCluster(double[], int, int, int, int) - Method in class jclustering.techniques.ClusteringTechnique
:   Provides a fast way to add a `double []` tac to a given cluster.

addTACtoCluster(Voxel, int) - Method in class jclustering.techniques.ClusteringTechnique
:   Provides a fast way to add a `Voxel` to a given cluster.

A C D E F G H I J K L M N P R S T U V X Y

- Overview
- Package
- Class
- Use
- Tree
- Deprecated
- Index
- Help

- Prev Letter
- Next Letter

- Frames
- No Frames

- All Classes
