# Supplementary material for: jClustering, an Open Framework for the Development of 4D Clustering Algorithms
Source: PLoS One. 2013 Aug 22;8(8):e70797. doi: 10.1371/journal.pone.0070797 (PMC3750055; doi:10.1371/journal.pone.0070797)
Supplement: File S1 — Public API for jClustering version 1.2.2. (ZIP) [file pone.0070797.s001.zip › index-files/index-12.html]

M-Index


JavaScript is disabled on your browser.


- Overview
- Package
- Class
- Use
- Tree
- Deprecated
- Index
- Help

- Prev Letter
- Next Letter

- Frames
- No Frames

- All Classes

A C D E F G H I J K L M N P R S T U V X Y 


## M

Mahalanobis - Class in jclustering.metrics
:   Implements a Mahanalnobis distance.

Mahalanobis() - Constructor for class jclustering.metrics.Mahalanobis


main(String[]) - Static method in class jclustering.JClustering\_
:   Main method for testing purposes.

makeConfig() - Method in class jclustering.metrics.ClusteringMetric
:   This function is called only once and returns the configuration panel
    that will be called by `ClusteringMetric.getConfig()` on each successive call.

makeConfig() - Method in class jclustering.metrics.PNorm


makeConfig() - Method in class jclustering.techniques.ICA


makeConfig() - Method in class jclustering.techniques.KMeans


makeConfig() - Method in class jclustering.techniques.LeaderFollower


makeConfig() - Method in class jclustering.techniques.PCA


makeConfig() - Method in class jclustering.techniques.SVD


MathUtils - Class in jclustering
:   Math helper class.

MathUtils() - Constructor for class jclustering.MathUtils

A C D E F G H I J K L M N P R S T U V X Y

- Overview
- Package
- Class
- Use
- Tree
- Deprecated
- Index
- Help

- Prev Letter
- Next Letter

- Frames
- No Frames

- All Classes
