# Supplementary material for: jClustering, an Open Framework for the Development of 4D Clustering Algorithms
Source: PLoS One. 2013 Aug 22;8(8):e70797. doi: 10.1371/journal.pone.0070797 (PMC3750055; doi:10.1371/journal.pone.0070797)
Supplement: File S1 — Public API for jClustering version 1.2.2. (ZIP) [file pone.0070797.s001.zip › index-files/index-14.html]

P-Index


JavaScript is disabled on your browser.


- Overview
- Package
- Class
- Use
- Tree
- Deprecated
- Index
- Help

- Prev Letter
- Next Letter

- Frames
- No Frames

- All Classes

A C D E F G H I J K L M N P R S T U V X Y 


## P

PACKAGE\_NAME - Static variable in class jclustering.Constants


PCA - Class in jclustering.techniques
:   Implements a PCA clustering according to
    this excellent
    guide.

PCA() - Constructor for class jclustering.techniques.PCA


PNorm - Class in jclustering.metrics
:   This `ClusteringMetric` implements a p-norm
    distance.

PNorm() - Constructor for class jclustering.metrics.PNorm


process() - Method in class jclustering.techniques.ClusteringTechnique
:   Performs the actual processing for this clustering technique.

process() - Method in class jclustering.techniques.ICA


process() - Method in class jclustering.techniques.KMeans


process() - Method in class jclustering.techniques.LeaderFollower


process() - Method in class jclustering.techniques.PCA


process() - Method in class jclustering.techniques.SampleTechnique


process() - Method in class jclustering.techniques.SVD

A C D E F G H I J K L M N P R S T U V X Y

- Overview
- Package
- Class
- Use
- Tree
- Deprecated
- Index
- Help

- Prev Letter
- Next Letter

- Frames
- No Frames

- All Classes
