# Supplementary material for: jClustering, an Open Framework for the Development of 4D Clustering Algorithms
Source: PLoS One. 2013 Aug 22;8(8):e70797. doi: 10.1371/journal.pone.0070797 (PMC3750055; doi:10.1371/journal.pone.0070797)
Supplement: File S1 — Public API for jClustering version 1.2.2. (ZIP) [file pone.0070797.s001.zip › index-files/index-15.html]

R-Index


JavaScript is disabled on your browser.


- Overview
- Package
- Class
- Use
- Tree
- Deprecated
- Index
- Help

- Prev Letter
- Next Letter

- Frames
- No Frames

- All Classes

A C D E F G H I J K L M N P R S T U V X Y 


## R

RealMatrix2IJ(RealMatrix, int[], ImagePlusHyp, boolean, String) - Static method in class jclustering.Utils
:   Transforms a `RealMatrix` object into a ImageJ image.

remove() - Method in class jclustering.ImagePlusHypIterator


resetMetricPanel(JPanel) - Static method in class jclustering.GUIUtils
:   Removes all the elements from the given panel and restores the default
    message.

resetTechPanel(JPanel) - Static method in class jclustering.GUIUtils
:   Removes all the elements from the given panel and restores the default
    message.

rmsd(double[], double[]) - Static method in class jclustering.MathUtils
:   Computes the root-mean-square deviation for the given TACs

RMSD - Class in jclustering.metrics
:   Root-mean-square deviation between two given TACs (data type
    `double[]`).

RMSD() - Constructor for class jclustering.metrics.RMSD


run(ImageProcessor) - Method in class jclustering.JClustering\_

A C D E F G H I J K L M N P R S T U V X Y

- Overview
- Package
- Class
- Use
- Tree
- Deprecated
- Index
- Help

- Prev Letter
- Next Letter

- Frames
- No Frames

- All Classes
