# Supplementary material for: jClustering, an Open Framework for the Development of 4D Clustering Algorithms
Source: PLoS One. 2013 Aug 22;8(8):e70797. doi: 10.1371/journal.pone.0070797 (PMC3750055; doi:10.1371/journal.pone.0070797)
Supplement: File S1 — Public API for jClustering version 1.2.2. (ZIP) [file pone.0070797.s001.zip › index-files/index-16.html]

S-Index


JavaScript is disabled on your browser.


- Overview
- Package
- Class
- Use
- Tree
- Deprecated
- Index
- Help

- Prev Letter
- Next Letter

- Frames
- No Frames

- All Classes

A C D E F G H I J K L M N P R S T U V X Y 


## S

SampleTechnique - Class in jclustering.techniques


SampleTechnique() - Constructor for class jclustering.techniques.SampleTechnique


save(String) - Method in class jclustering.FileSaver
:   Saves the data in the specified path in the specified format.

setCentroid(double[]) - Method in class jclustering.Cluster
:   Sets this cluster's centroid.

setMetric(ClusteringMetric) - Method in class jclustering.techniques.ClusteringTechnique
:   Sets the current ClusteringMetric

setPanel(JPanel, JPanel, JFrame) - Static method in class jclustering.GUIUtils
:   Sets a panel inside a panel and updates the size of the containing
    window.

setup(String, ImagePlus) - Method in class jclustering.JClustering\_


setup(ImagePlusHyp) - Method in class jclustering.metrics.ClusteringMetric
:   Setup method, as the constructor will always be called empty.

setup(ImagePlusHyp) - Method in class jclustering.techniques.ClusteringTechnique
:   Setup method, as the constructor will always be called empty.

setVoxel(ImageStack, int, int, int, double) - Static method in class jclustering.Utils
:   Provides a handy method for setting voxel values using slices as
    z-indexes (which are 0-based).

size() - Method in class jclustering.Cluster


skip\_noisy(boolean) - Method in class jclustering.metrics.ClusteringMetric
:   Sets the skip\_noisy variable for this metric.

skipNoisy(boolean) - Method in class jclustering.techniques.ClusteringTechnique
:   Changes this techniques's behavior with respect to noisy voxels.

slice - Variable in class jclustering.Voxel
:   Slice (1-based) for the voxel.

smooth(double[]) - Static method in class jclustering.MathUtils
:   Smooths the given TAC using a 5-point filtering.

SVD - Class in jclustering.techniques
:   Implements a SVD on the original image matrix.

SVD() - Constructor for class jclustering.techniques.SVD

A C D E F G H I J K L M N P R S T U V X Y

- Overview
- Package
- Class
- Use
- Tree
- Deprecated
- Index
- Help

- Prev Letter
- Next Letter

- Frames
- No Frames

- All Classes
