# Supplementary material for: jClustering, an Open Framework for the Development of 4D Clustering Algorithms
Source: PLoS One. 2013 Aug 22;8(8):e70797. doi: 10.1371/journal.pone.0070797 (PMC3750055; doi:10.1371/journal.pone.0070797)
Supplement: File S1 — Public API for jClustering version 1.2.2. (ZIP) [file pone.0070797.s001.zip › index-files/index-2.html]

C-Index


JavaScript is disabled on your browser.


- Overview
- Package
- Class
- Use
- Tree
- Deprecated
- Index
- Help

- Prev Letter
- Next Letter

- Frames
- No Frames

- All Classes

A C D E F G H I J K L M N P R S T U V X Y 


## C

Cluster - Class in jclustering
:   Implements a cluster class.

Cluster() - Constructor for class jclustering.Cluster
:   Public constructor.

Cluster(double[]) - Constructor for class jclustering.Cluster
:   Public constructor with a pre-defined centroid.

Cluster(double[], int, int, int) - Constructor for class jclustering.Cluster
:   Public constructor with a pre-defined centroid and coordinates for it.

Cluster(Voxel) - Constructor for class jclustering.Cluster
:   Provides a shortcut for the public constructor with parameters using a
    `Voxel` to initialize the parameters.

ClusteringMetric - Class in jclustering.metrics
:   This abstract class provides a template with the basic functions that a
    metric should implement, specially the distance(double [], double[]) method.

ClusteringMetric() - Constructor for class jclustering.metrics.ClusteringMetric


ClusteringTechnique - Class in jclustering.techniques
:   This superclass should be extended by all user-implemented clustering
    techniques.

ClusteringTechnique() - Constructor for class jclustering.techniques.ClusteringTechnique


componentHidden(ComponentEvent) - Method in class jclustering.JClustering\_


componentMoved(ComponentEvent) - Method in class jclustering.JClustering\_


componentResized(ComponentEvent) - Method in class jclustering.JClustering\_


componentShown(ComponentEvent) - Method in class jclustering.JClustering\_


compute() - Method in class jclustering.techniques.ClusteringTechnique
:   This helper method is the method called from the main class.

Constants - Class in jclustering
:   Define the necessary constants.

Constants() - Constructor for class jclustering.Constants


Correlation - Class in jclustering.metrics
:   Classical correlation score between two given TACs (data type
    `double[]`).

Correlation() - Constructor for class jclustering.metrics.Correlation


createButton(String, ActionListener) - Static method in class jclustering.GUIUtils
:   Creates a `Button` with a given label used as name and an
    ActionListener.

createButton(String, String, ActionListener) - Static method in class jclustering.GUIUtils
:   Creates a `Button` with a given text, a given name and a given
    ActionListener.

createChoices(String, String[], ItemListener) - Static method in class jclustering.GUIUtils
:   Creates a JComboBox dropdown list.

createChoices(String, ArrayList<String>, ItemListener) - Static method in class jclustering.GUIUtils
:   Creates a JComboBox dropdown list.

createImagePlus(int, int, int, int) - Static method in class jclustering.Utils
:   Creates an empty ImagePlus with the given dimensions.

createJButton(String, String, ActionListener) - Static method in class jclustering.GUIUtils
:   Creates a `JButton` with a given text, a given name and a given
    ActionListener.

createJButton(String, ActionListener) - Static method in class jclustering.GUIUtils
:   Creates a `Button` with a given label used as name and an
    ActionListener.

createJLabel(String, String) - Static method in class jclustering.GUIUtils
:   Creates a JLabel with a given text and a given help message

createJPanel(String, LayoutManager, ComponentListener) - Static method in class jclustering.GUIUtils
:   Auxiliar method for creating a named JPanel object.

createJPanel(String, ComponentListener) - Static method in class jclustering.GUIUtils
:   Auxiliar method for creating a named JPanel object.

createJTextField(String, int, FocusListener) - Static method in class jclustering.GUIUtils
:   Creates a new JTextField with the given name, integer value and adds
    a FocusListener.

createJTextField(String, double, FocusListener) - Static method in class jclustering.GUIUtils
:   Creates a new JTextField with the given name, double value and adds
    a FocusListener.

A C D E F G H I J K L M N P R S T U V X Y

- Overview
- Package
- Class
- Use
- Tree
- Deprecated
- Index
- Help

- Prev Letter
- Next Letter

- Frames
- No Frames

- All Classes
