# Supplementary material for: jClustering, an Open Framework for the Development of 4D Clustering Algorithms
Source: PLoS One. 2013 Aug 22;8(8):e70797. doi: 10.1371/journal.pone.0070797 (PMC3750055; doi:10.1371/journal.pone.0070797)
Supplement: File S1 — Public API for jClustering version 1.2.2. (ZIP) [file pone.0070797.s001.zip › index-files/index-5.html]

F-Index


JavaScript is disabled on your browser.


- Overview
- Package
- Class
- Use
- Tree
- Deprecated
- Index
- Help

- Prev Letter
- Next Letter

- Frames
- No Frames

- All Classes

A C D E F G H I J K L M N P R S T U V X Y 


## F

FileSaver - Class in jclustering
:   Class for file saving.

FileSaver(String, ArrayList<Cluster>, double[][], String[]) - Constructor for class jclustering.FileSaver
:   Constructor

focusGained(FocusEvent) - Method in class jclustering.metrics.PNorm


focusGained(FocusEvent) - Method in class jclustering.techniques.ICA


focusGained(FocusEvent) - Method in class jclustering.techniques.KMeans


focusGained(FocusEvent) - Method in class jclustering.techniques.LeaderFollower


focusLost(FocusEvent) - Method in class jclustering.metrics.PNorm


focusLost(FocusEvent) - Method in class jclustering.techniques.ICA


focusLost(FocusEvent) - Method in class jclustering.techniques.KMeans


focusLost(FocusEvent) - Method in class jclustering.techniques.LeaderFollower

A C D E F G H I J K L M N P R S T U V X Y

- Overview
- Package
- Class
- Use
- Tree
- Deprecated
- Index
- Help

- Prev Letter
- Next Letter

- Frames
- No Frames

- All Classes
