# Supplementary material for: jClustering, an Open Framework for the Development of 4D Clustering Algorithms
Source: PLoS One. 2013 Aug 22;8(8):e70797. doi: 10.1371/journal.pone.0070797 (PMC3750055; doi:10.1371/journal.pone.0070797)
Supplement: File S1 — Public API for jClustering version 1.2.2. (ZIP) [file pone.0070797.s001.zip › index-files/index-6.html]

G-Index


JavaScript is disabled on your browser.


- Overview
- Package
- Class
- Use
- Tree
- Deprecated
- Index
- Help

- Prev Letter
- Next Letter

- Frames
- No Frames

- All Classes

A C D E F G H I J K L M N P R S T U V X Y 


## G

getAdditionalInfo() - Method in class jclustering.techniques.ClusteringTechnique
:   Returns additional information generated by this technique in a String
    array.

getAllMetrics() - Static method in class jclustering.Utils
:   Finds all the classes that extend the ClusteringMetric superclass and
    returns a list of their names.

getAllTechniques() - Static method in class jclustering.Utils
:   Finds all the classes that extend the ClusteringTechnique superclass and
    returns a list of their names.

getCentroid() - Method in class jclustering.Cluster


getCloserClusterIndex(double[]) - Method in class jclustering.techniques.ClusteringTechnique
:   Finds the cluster with a centroid as close as possible to the given TAC.

getClusterAt(int) - Method in class jclustering.techniques.ClusteringTechnique
:   Provides a safe way to get the `Cluster` at the `index`
    position.

getClusteringMetric(String, ImagePlusHyp) - Static method in class jclustering.Utils
:   Builds a new instance for a `ClusteringMetric` object and returns
    it.

getClusteringTechnique(String, ImagePlusHyp, boolean) - Static method in class jclustering.Utils
:   Builds a new instance for a `ClusteringTechnique` object and
    returns it.

getClusters() - Method in class jclustering.techniques.ClusteringTechnique


getClusterTAC() - Method in class jclustering.Cluster


getConfig() - Method in class jclustering.metrics.ClusteringMetric
:   Builds a configuration `Panel` that will provide all the necessary
    interfaces for the technique configuration.

getConfig() - Method in class jclustering.techniques.ClusteringTechnique
:   Builds a configuration `Panel` that will provide all the necessary
    interfaces for the technique configuration.

getCoordinates() - Method in class jclustering.Cluster


getMaxIndex(double[]) - Static method in class jclustering.MathUtils
:   Returns the index for the maximum value of the array.

getMetric() - Method in class jclustering.techniques.ClusteringTechnique


getMetricList(ClusteringTechnique, ImagePlusHyp) - Static method in class jclustering.GUIUtils
:   Returns a `JComboBox` of `ClusteringMetric` objects to be used
    inside the `ClusteringTechnique` `t`.

getName() - Method in class jclustering.metrics.ClusteringMetric


getName() - Method in class jclustering.techniques.ClusteringTechnique


getName(T) - Static method in class jclustering.Utils
:   Returns the name of a given class, without the package name

getPeakMean() - Method in class jclustering.Cluster


getPeakStats() - Method in class jclustering.Cluster


getPeakStdev() - Method in class jclustering.Cluster


getTAC(int, int, int) - Method in class jclustering.ImagePlusHyp
:   Gets the time-activity curve (dixel, after "dynamic pixel") for the
    given coordinates.

getTimeVector() - Method in class jclustering.TimeVectorReader
:   Reads the data provided in the constructor and returns the appropriate
    time vector array.

GUIUtils - Class in jclustering
:   Auxiliar class with static utility methods for GUI creation

GUIUtils() - Constructor for class jclustering.GUIUtils

A C D E F G H I J K L M N P R S T U V X Y

- Overview
- Package
- Class
- Use
- Tree
- Deprecated
- Index
- Help

- Prev Letter
- Next Letter

- Frames
- No Frames

- All Classes
