# Supplementary material for: jClustering, an Open Framework for the Development of 4D Clustering Algorithms
Source: PLoS One. 2013 Aug 22;8(8):e70797. doi: 10.1371/journal.pone.0070797 (PMC3750055; doi:10.1371/journal.pone.0070797)
Supplement: File S1 — Public API for jClustering version 1.2.2. (ZIP) [file pone.0070797.s001.zip › index-files/index-8.html]

I-Index


JavaScript is disabled on your browser.


- Overview
- Package
- Class
- Use
- Tree
- Deprecated
- Index
- Help

- Prev Letter
- Next Letter

- Frames
- No Frames

- All Classes

A C D E F G H I J K L M N P R S T U V X Y 


## I

ICA - Class in jclustering.techniques
:   Implements an Independent Component Analysis on the image data.

ICA() - Constructor for class jclustering.techniques.ICA


ImagePlusHyp - Class in jclustering
:   This class extends `ImagePlus` in order to add a handy `ImagePlusHyp.getTAC(int, int, int)`
    method that allows to easily grab time-activity curves.

ImagePlusHyp(ImagePlus) - Constructor for class jclustering.ImagePlusHyp
:   Creates a new ImagePlusHyp using a general ImagePlus.

ImagePlusHypIterator - Class in jclustering
:   Provides an `Iterator` for an `ImagePlusHyp` object.

ImagePlusHypIterator(ImagePlusHyp) - Constructor for class jclustering.ImagePlusHypIterator
:   Public constructor.

init() - Method in class jclustering.metrics.ClusteringMetric
:   If the metric needs some previous computations, it should override
    this method.

init() - Method in class jclustering.metrics.Correlation


init() - Method in class jclustering.metrics.Mahalanobis


init() - Method in class jclustering.techniques.ClusteringTechnique
:   Initializes the clusters ArrayList.

isEmpty() - Method in class jclustering.Cluster


isNoise(double[]) - Method in class jclustering.ImagePlusHyp


isNoise(double[]) - Method in class jclustering.metrics.ClusteringMetric
:   Ease of access for the `ImagePlusHyp.isNoise(double[])` method.

isNoise(Voxel) - Method in class jclustering.metrics.ClusteringMetric
:   Ease of access for the `ImagePlusHyp.isNoise(double[])` method.

isNoise(double[]) - Method in class jclustering.techniques.ClusteringTechnique
:   Helper method.

isNoise(Voxel) - Method in class jclustering.techniques.ClusteringTechnique
:   Helper method.

itemStateChanged(ItemEvent) - Method in class jclustering.JClustering\_


itemStateChanged(ItemEvent) - Method in class jclustering.metrics.ClusteringMetric


itemStateChanged(ItemEvent) - Method in class jclustering.techniques.ClusteringTechnique


itemStateChanged(ItemEvent) - Method in class jclustering.techniques.ICA


itemStateChanged(ItemEvent) - Method in class jclustering.techniques.KMeans


itemStateChanged(ItemEvent) - Method in class jclustering.techniques.LeaderFollower


itemStateChanged(ItemEvent) - Method in class jclustering.techniques.PCA


itemStateChanged(ItemEvent) - Method in class jclustering.techniques.SampleTechnique


itemStateChanged(ItemEvent) - Method in class jclustering.techniques.SVD


iterator() - Method in class jclustering.ImagePlusHyp

A C D E F G H I J K L M N P R S T U V X Y

- Overview
- Package
- Class
- Use
- Tree
- Deprecated
- Index
- Help

- Prev Letter
- Next Letter

- Frames
- No Frames

- All Classes
