# Supplementary material for: jClustering, an Open Framework for the Development of 4D Clustering Algorithms
Source: PLoS One. 2013 Aug 22;8(8):e70797. doi: 10.1371/journal.pone.0070797 (PMC3750055; doi:10.1371/journal.pone.0070797)
Supplement: File S1 — Public API for jClustering version 1.2.2. (ZIP) [file pone.0070797.s001.zip › jclustering/class-use/Cluster.html]

Uses of Class jclustering.Cluster


JavaScript is disabled on your browser.


- Overview
- Package
- Class
- Use
- Tree
- Deprecated
- Index
- Help

- Prev
- Next

- Frames
- No Frames

- All Classes

## Uses of Class jclustering.Cluster

- Packages that use Cluster

  | Package | Description |
  |  |  |
  | --- | --- |
  | jclustering |  |
  | jclustering.techniques |  |
- - ### Uses of Cluster in jclustering

    Constructor parameters in jclustering with type arguments of type Cluster

    | Constructor and Description |
    |  |
    | --- |
    | `FileSaver(java.lang.String format, java.util.ArrayList<Cluster> clusters, double[][] t, java.lang.String[] additionalInfo)` Constructor |
  - ### Uses of Cluster in jclustering.techniques

    Methods in jclustering.techniques that return Cluster

    | Modifier and Type | Method and Description |
    |  |  |
    | --- | --- |
    | `Cluster` | ClusteringTechnique.`addCluster(double[] tac)` Creates a new cluster with the `double [] tac` as the centroid, adds it to the cluster ArrayList and returns it. |
    | `Cluster` | ClusteringTechnique.`getClusterAt(int index)` Provides a safe way to get the `Cluster` at the `index` position. |

    Methods in jclustering.techniques that return types with arguments of type Cluster

    | Modifier and Type | Method and Description |
    |  |  |
    | --- | --- |
    | `java.util.ArrayList<Cluster>` | ClusteringTechnique.`getClusters()` |

- Overview
- Package
- Class
- Use
- Tree
- Deprecated
- Index
- Help

- Prev
- Next

- Frames
- No Frames

- All Classes
