# Supplementary material for: jClustering, an Open Framework for the Development of 4D Clustering Algorithms
Source: PLoS One. 2013 Aug 22;8(8):e70797. doi: 10.1371/journal.pone.0070797 (PMC3750055; doi:10.1371/journal.pone.0070797)
Supplement: File S1 — Public API for jClustering version 1.2.2. (ZIP) [file pone.0070797.s001.zip › jclustering/class-use/ImagePlusHyp.html]

Uses of Class jclustering.ImagePlusHyp


JavaScript is disabled on your browser.


- Overview
- Package
- Class
- Use
- Tree
- Deprecated
- Index
- Help

- Prev
- Next

- Frames
- No Frames

- All Classes

## Uses of Class jclustering.ImagePlusHyp

- Packages that use ImagePlusHyp

  | Package | Description |
  |  |  |
  | --- | --- |
  | jclustering |  |
  | jclustering.metrics |  |
  | jclustering.techniques |  |
- - ### Uses of ImagePlusHyp in jclustering

    Methods in jclustering with parameters of type ImagePlusHyp

    | Modifier and Type | Method and Description |
    |  |  |
    | --- | --- |
    | `static ClusteringMetric` | Utils.`getClusteringMetric(java.lang.String name, ImagePlusHyp ip)` Builds a new instance for a `ClusteringMetric` object and returns it. |
    | `static ClusteringTechnique` | Utils.`getClusteringTechnique(java.lang.String name, ImagePlusHyp ip, boolean skip_noisy)` Builds a new instance for a `ClusteringTechnique` object and returns it. |
    | `static javax.swing.JComboBox` | GUIUtils.`getMetricList(ClusteringTechnique t, ImagePlusHyp ip)` Returns a `JComboBox` of `ClusteringMetric` objects to be used inside the `ClusteringTechnique` `t`. |
    | `static ij.ImagePlus` | Utils.`RealMatrix2IJ(org.apache.commons.math3.linear.RealMatrix rm, int[] dim, ImagePlusHyp ip, boolean skip_noisy, java.lang.String name)` Transforms a `RealMatrix` object into a ImageJ image. |

    Constructors in jclustering with parameters of type ImagePlusHyp

    | Constructor and Description |
    |  |
    | --- |
    | `ImagePlusHypIterator(ImagePlusHyp ip)` Public constructor. |
  - ### Uses of ImagePlusHyp in jclustering.metrics

    Methods in jclustering.metrics with parameters of type ImagePlusHyp

    | Modifier and Type | Method and Description |
    |  |  |
    | --- | --- |
    | `void` | ClusteringMetric.`setup(ImagePlusHyp ip)` Setup method, as the constructor will always be called empty. |
  - ### Uses of ImagePlusHyp in jclustering.techniques

    Methods in jclustering.techniques with parameters of type ImagePlusHyp

    | Modifier and Type | Method and Description |
    |  |  |
    | --- | --- |
    | `void` | ClusteringTechnique.`setup(ImagePlusHyp ip)` Setup method, as the constructor will always be called empty. |

- Overview
- Package
- Class
- Use
- Tree
- Deprecated
- Index
- Help

- Prev
- Next

- Frames
- No Frames

- All Classes
