# Supplementary material for: jClustering, an Open Framework for the Development of 4D Clustering Algorithms
Source: PLoS One. 2013 Aug 22;8(8):e70797. doi: 10.1371/journal.pone.0070797 (PMC3750055; doi:10.1371/journal.pone.0070797)
Supplement: File S1 — Public API for jClustering version 1.2.2. (ZIP) [file pone.0070797.s001.zip › jclustering/class-use/Voxel.html]

Uses of Class jclustering.Voxel


JavaScript is disabled on your browser.


- Overview
- Package
- Class
- Use
- Tree
- Deprecated
- Index
- Help

- Prev
- Next

- Frames
- No Frames

- All Classes

## Uses of Class jclustering.Voxel

- Packages that use Voxel

  | Package | Description |
  |  |  |
  | --- | --- |
  | jclustering |  |
  | jclustering.metrics |  |
  | jclustering.techniques |  |
- - ### Uses of Voxel in jclustering

    Methods in jclustering that return Voxel

    | Modifier and Type | Method and Description |
    |  |  |
    | --- | --- |
    | `Voxel` | ImagePlusHypIterator.`next()` |

    Methods in jclustering that return types with arguments of type Voxel

    | Modifier and Type | Method and Description |
    |  |  |
    | --- | --- |
    | `java.util.Iterator<Voxel>` | ImagePlusHyp.`iterator()` |

    Methods in jclustering with parameters of type Voxel

    | Modifier and Type | Method and Description |
    |  |  |
    | --- | --- |
    | `void` | Cluster.`add(Voxel v)` Provides a shortcut to the `Cluster.add(double[], int, int, int)` method using a `Voxel`. |

    Constructors in jclustering with parameters of type Voxel

    | Constructor and Description |
    |  |
    | --- |
    | `Cluster(Voxel v)` Provides a shortcut for the public constructor with parameters using a `Voxel` to initialize the parameters. |
  - ### Uses of Voxel in jclustering.metrics

    Methods in jclustering.metrics with parameters of type Voxel

    | Modifier and Type | Method and Description |
    |  |  |
    | --- | --- |
    | `double` | ClusteringMetric.`distance(Voxel v, double[] centroid)` Provides a shortcut for computing the distance between a `Voxel` and any TAC. |
    | `boolean` | ClusteringMetric.`isNoise(Voxel v)` Ease of access for the `ImagePlusHyp.isNoise(double[])` method. |
  - ### Uses of Voxel in jclustering.techniques

    Methods in jclustering.techniques with parameters of type Voxel

    | Modifier and Type | Method and Description |
    |  |  |
    | --- | --- |
    | `void` | ClusteringTechnique.`addTACtoCluster(Voxel v, int cluster)` Provides a fast way to add a `Voxel` to a given cluster. |
    | `boolean` | ClusteringTechnique.`isNoise(Voxel v)` Helper method. |

- Overview
- Package
- Class
- Use
- Tree
- Deprecated
- Index
- Help

- Prev
- Next

- Frames
- No Frames

- All Classes
