# Supplementary material for: jClustering, an Open Framework for the Development of 4D Clustering Algorithms
Source: PLoS One. 2013 Aug 22;8(8):e70797. doi: 10.1371/journal.pone.0070797 (PMC3750055; doi:10.1371/journal.pone.0070797)
Supplement: File S1 — Public API for jClustering version 1.2.2. (ZIP) [file pone.0070797.s001.zip › jclustering/Cluster.html]

Cluster


JavaScript is disabled on your browser.


- Overview
- Package
- Class
- Use
- Tree
- Deprecated
- Index
- Help

- Prev Class
- Next Class

- Frames
- No Frames

- All Classes

- Summary:
- Nested |
- Field |
- Constr |
- Method

- Detail:
- Field |
- Constr |
- Method


jclustering

## Class Cluster

- java.lang.Object
- - jclustering.Cluster

- ---

    

  ```
  public class Cluster
  extends java.lang.Object
  ```

  Implements a cluster class. A cluster is defined by a centroid (a
  `double []` array) and by the mean TAC of the voxels assigned to this
  cluster. Every other elements from this class serve as helpers for latter
  calculations.

  Author:
  :   José María Mateos.

- - ### Constructor Summary

    Constructors

    | Constructor and Description |
    | `Cluster()` Public constructor. |
    | `Cluster(double[] centroid)` Public constructor with a pre-defined centroid. |
    | `Cluster(double[] centroid, int x, int y, int slice)` Public constructor with a pre-defined centroid and coordinates for it. |
    | `Cluster(Voxel v)` Provides a shortcut for the public constructor with parameters using a `Voxel` to initialize the parameters. |
  - ### Method Summary

    Methods

    | Modifier and Type | Method and Description |
    | `void` | `add(double[] data, int x, int y, int slice)` Adds given data to cluster. |
    | `void` | `add(Voxel v)` Provides a shortcut to the `add(double[], int, int, int)` method using a `Voxel`. |
    | `double[]` | `getCentroid()` |
    | `double[]` | `getClusterTAC()` |
    | `java.util.ArrayList<java.lang.Integer[]>` | `getCoordinates()` |
    | `double` | `getPeakMean()` |
    | `org.apache.commons.math3.stat.descriptive.SummaryStatistics` | `getPeakStats()` |
    | `double` | `getPeakStdev()` |
    | `boolean` | `isEmpty()` |
    | `void` | `setCentroid(double[] centroid)` Sets this cluster's centroid. |
    | `int` | `size()` |

    - ### Methods inherited from class java.lang.Object

      `equals, getClass, hashCode, notify, notifyAll, toString, wait, wait, wait`

- - ### Constructor Detail


    - #### Cluster

      ```
      public Cluster()
      ```

      Public constructor. Does nothing by default, just initializes the
      internal containers. A cluster created this way modifies its centroid.


    - #### Cluster

      ```
      public Cluster(double[] centroid)
      ```

      Public constructor with a pre-defined centroid. A cluster initialized
      this way does not modify its centroid.

      Parameters:
      :   `centroid` - Centroid vector.


    - #### Cluster

      ```
      public Cluster(double[] centroid,
             int x,
             int y,
             int slice)
      ```

      Public constructor with a pre-defined centroid and coordinates for it.
      If this method is used as a constructor, it is assumed that the the
      centroid for this cluster will be modified with each new TAC that is
      added to this object.

      Parameters:
      :   `centroid` - Centroid vector.
      :   `x` - X-coordinate for the centroid TAC.
      :   `y` - Y-coordinate for the centroid TAC.
      :   `slice` - Slice (1-based) for the centroid TAC. TAC.


    - #### Cluster

      ```
      public Cluster(Voxel v)
      ```

      Provides a shortcut for the public constructor with parameters using a
      `Voxel` to initialize the parameters. A cluster initialized this
      way modifies its centroid.

      Parameters:
      :   `v` - The voxel to be used as initial centroid.
  - ### Method Detail


    - #### getCoordinates

      ```
      public java.util.ArrayList<java.lang.Integer[]> getCoordinates()
      ```

      Returns:
      :   The coordinates of pixels added to this cluster


    - #### getCentroid

      ```
      public double[] getCentroid()
      ```

      Returns:
      :   The current centroid data.


    - #### setCentroid

      ```
      public void setCentroid(double[] centroid)
      ```

      Sets this cluster's centroid.

      Parameters:
      :   `centroid` - The new centroid.


    - #### getClusterTAC

      ```
      public double[] getClusterTAC()
      ```

      Returns:
      :   The current cluster TAC.


    - #### size

      ```
      public int size()
      ```

      Returns:
      :   The number of pixels inside this cluster.


    - #### add

      ```
      public void add(double[] data,
             int x,
             int y,
             int slice)
      ```

      Adds given data to cluster. It can work in two different ways:
      - It can modify this cluster's centroid as new voxels are added.- The centroid can remain static and the news voxels are added
          independently, forming a new TAC.The second way is the standard way of creating a new cluster, where the
      centroid serves as the reference for the distances. In any case, as there
      are some approaches in which the centroid is built along the way, the
      `Cluster` constructor will cause this cluster to modify its
      centroid if the coordinates for the first voxel are provided.
      This method always uses the default behavior used at creation
      time.

      Parameters:
      :   `data` - Dynamic data to be added
      :   `x` - X-coordinate for added TAC.
      :   `y` - Y-coordinate for added TAC.
      :   `slice` - Slice (1-based) for added TAC.


    - #### add

      ```
      public void add(Voxel v)
      ```

      Provides a shortcut to the `add(double[], int, int, int)`
      method using a `Voxel`.

      Parameters:
      :   `v` - The Voxel to be added.


    - #### getPeakStats

      ```
      public org.apache.commons.math3.stat.descriptive.SummaryStatistics getPeakStats()
      ```

      Returns:
      :   A `SummaryStatistics` object with the peak amplitudes data.


    - #### getPeakMean

      ```
      public double getPeakMean()
      ```

      Returns:
      :   Mean peak value for all TACs in cluster.


    - #### getPeakStdev

      ```
      public double getPeakStdev()
      ```

      Returns:
      :   Standard deviation for peak values for all TACs in cluster.


    - #### isEmpty

      ```
      public boolean isEmpty()
      ```

      Returns:
      :   true if the given cluster is empty


- Overview
- Package
- Class
- Use
- Tree
- Deprecated
- Index
- Help

- Prev Class
- Next Class

- Frames
- No Frames

- All Classes

- Summary:
- Nested |
- Field |
- Constr |
- Method

- Detail:
- Field |
- Constr |
- Method
