# Supplementary material for: jClustering, an Open Framework for the Development of 4D Clustering Algorithms
Source: PLoS One. 2013 Aug 22;8(8):e70797. doi: 10.1371/journal.pone.0070797 (PMC3750055; doi:10.1371/journal.pone.0070797)
Supplement: File S1 — Public API for jClustering version 1.2.2. (ZIP) [file pone.0070797.s001.zip › jclustering/Constants.html]

Constants


JavaScript is disabled on your browser.


- Overview
- Package
- Class
- Use
- Tree
- Deprecated
- Index
- Help

- Prev Class
- Next Class

- Frames
- No Frames

- All Classes

- Summary:
- Nested |
- Field |
- Constr |
- Method

- Detail:
- Field |
- Constr |
- Method


jclustering

## Class Constants

- java.lang.Object
- - jclustering.Constants

- ---

    

  ```
  public class Constants
  extends java.lang.Object
  ```

  Define the necessary constants. That includes physical constants or
  configuration parameters.

  Author:
  :   José María Mateos

- - ### Field Summary

    Fields

    | Modifier and Type | Field and Description |
    | `static java.lang.String` | `NO_METRIC` |
    | `static java.lang.String` | `NO_TECHNIQUE` |
    | `static java.lang.String` | `PACKAGE_NAME` |
    | `static java.lang.String` | `VERSION` |
  - ### Constructor Summary

    Constructors

    | Constructor and Description |
    | `Constants()` |
  - ### Method Summary

    - ### Methods inherited from class java.lang.Object

      `equals, getClass, hashCode, notify, notifyAll, toString, wait, wait, wait`

- - ### Field Detail


    - #### PACKAGE\_NAME

      ```
      public static final java.lang.String PACKAGE_NAME
      ```

      See Also:
      :   Constant Field Values


    - #### NO\_METRIC

      ```
      public static final java.lang.String NO_METRIC
      ```

      See Also:
      :   Constant Field Values


    - #### NO\_TECHNIQUE

      ```
      public static final java.lang.String NO_TECHNIQUE
      ```

      See Also:
      :   Constant Field Values


    - #### VERSION

      ```
      public static final java.lang.String VERSION
      ```

      See Also:
      :   Constant Field Values
  - ### Constructor Detail


    - #### Constants

      ```
      public Constants()
      ```


- Overview
- Package
- Class
- Use
- Tree
- Deprecated
- Index
- Help

- Prev Class
- Next Class

- Frames
- No Frames

- All Classes

- Summary:
- Nested |
- Field |
- Constr |
- Method

- Detail:
- Field |
- Constr |
- Method
