# Supplementary material for: jClustering, an Open Framework for the Development of 4D Clustering Algorithms
Source: PLoS One. 2013 Aug 22;8(8):e70797. doi: 10.1371/journal.pone.0070797 (PMC3750055; doi:10.1371/journal.pone.0070797)
Supplement: File S1 — Public API for jClustering version 1.2.2. (ZIP) [file pone.0070797.s001.zip › jclustering/FileSaver.html]

FileSaver


JavaScript is disabled on your browser.


- Overview
- Package
- Class
- Use
- Tree
- Deprecated
- Index
- Help

- Prev Class
- Next Class

- Frames
- No Frames

- All Classes

- Summary:
- Nested |
- Field |
- Constr |
- Method

- Detail:
- Field |
- Constr |
- Method


jclustering

## Class FileSaver

- java.lang.Object
- - jclustering.FileSaver

- ---

    

  ```
  public class FileSaver
  extends java.lang.Object
  ```

  Class for file saving. It accepts several formats as output. Logs result
  of writing operation using the `IJ.log` functionality.

  Author:
  :   José María Mateos.

- - ### Constructor Summary

    Constructors

    | Constructor and Description |
    | `FileSaver(java.lang.String format, java.util.ArrayList<Cluster> clusters, double[][] t, java.lang.String[] additionalInfo)` Constructor |
  - ### Method Summary

    Methods

    | Modifier and Type | Method and Description |
    | `void` | `save(java.lang.String path)` Saves the data in the specified path in the specified format. |

    - ### Methods inherited from class java.lang.Object

      `equals, getClass, hashCode, notify, notifyAll, toString, wait, wait, wait`

- - ### Constructor Detail


    - #### FileSaver

      ```
      public FileSaver(java.lang.String format,
               java.util.ArrayList<Cluster> clusters,
               double[][] t,
               java.lang.String[] additionalInfo)
      ```

      Constructor

      Parameters:
      :   `format` - File saving format.
      :   `clusters` - The TAC data to be saved.
      :   `t` - Time vector.
      :   `additionalInfo` - Additional information provided.
  - ### Method Detail


    - #### save

      ```
      public void save(java.lang.String path)
      ```

      Saves the data in the specified path in the specified format.

      Parameters:
      :   `path` - The path for the file to be saved. May include the file
          name.


- Overview
- Package
- Class
- Use
- Tree
- Deprecated
- Index
- Help

- Prev Class
- Next Class

- Frames
- No Frames

- All Classes

- Summary:
- Nested |
- Field |
- Constr |
- Method

- Detail:
- Field |
- Constr |
- Method
