# Supplementary material for: jClustering, an Open Framework for the Development of 4D Clustering Algorithms
Source: PLoS One. 2013 Aug 22;8(8):e70797. doi: 10.1371/journal.pone.0070797 (PMC3750055; doi:10.1371/journal.pone.0070797)
Supplement: File S1 — Public API for jClustering version 1.2.2. (ZIP) [file pone.0070797.s001.zip › jclustering/GUIUtils.html]

GUIUtils


JavaScript is disabled on your browser.


- Overview
- Package
- Class
- Use
- Tree
- Deprecated
- Index
- Help

- Prev Class
- Next Class

- Frames
- No Frames

- All Classes

- Summary:
- Nested |
- Field |
- Constr |
- Method

- Detail:
- Field |
- Constr |
- Method


jclustering

## Class GUIUtils

- java.lang.Object
- - jclustering.GUIUtils

- ---

    

  ```
  public class GUIUtils
  extends java.lang.Object
  ```

  Auxiliar class with static utility methods for GUI creation

  Author:
  :   José María Mateos.

- - ### Constructor Summary

    Constructors

    | Constructor and Description |
    | `GUIUtils()` |
  - ### Method Summary

    Methods

    | Modifier and Type | Method and Description |
    | `static java.awt.Button` | `createButton(java.lang.String name, java.awt.event.ActionListener a)` Creates a `Button` with a given label used as name and an ActionListener. |
    | `static java.awt.Button` | `createButton(java.lang.String text, java.lang.String name, java.awt.event.ActionListener a)` Creates a `Button` with a given text, a given name and a given ActionListener. |
    | `static javax.swing.JComboBox` | `createChoices(java.lang.String name, java.util.ArrayList<java.lang.String> values, java.awt.event.ItemListener i)` Creates a JComboBox dropdown list. |
    | `static javax.swing.JComboBox` | `createChoices(java.lang.String name, java.lang.String[] values, java.awt.event.ItemListener i)` Creates a JComboBox dropdown list. |
    | `static javax.swing.JButton` | `createJButton(java.lang.String text, java.awt.event.ActionListener a)` Creates a `Button` with a given label used as name and an ActionListener. |
    | `static javax.swing.JButton` | `createJButton(java.lang.String text, java.lang.String name, java.awt.event.ActionListener a)` Creates a `JButton` with a given text, a given name and a given ActionListener. |
    | `static javax.swing.JLabel` | `createJLabel(java.lang.String text, java.lang.String help)` Creates a JLabel with a given text and a given help message |
    | `static javax.swing.JPanel` | `createJPanel(java.lang.String name, java.awt.event.ComponentListener cl)` Auxiliar method for creating a named JPanel object. |
    | `static javax.swing.JPanel` | `createJPanel(java.lang.String name, java.awt.LayoutManager lm, java.awt.event.ComponentListener cl)` Auxiliar method for creating a named JPanel object. |
    | `static javax.swing.JTextField` | `createJTextField(java.lang.String name, double value, java.awt.event.FocusListener fl)` Creates a new JTextField with the given name, double value and adds a FocusListener. |
    | `static javax.swing.JTextField` | `createJTextField(java.lang.String name, int value, java.awt.event.FocusListener fl)` Creates a new JTextField with the given name, integer value and adds a FocusListener. |
    | `static javax.swing.JComboBox` | `getMetricList(ClusteringTechnique t, ImagePlusHyp ip)` Returns a `JComboBox` of `ClusteringMetric` objects to be used inside the `ClusteringTechnique` `t`. |
    | `static void` | `resetMetricPanel(javax.swing.JPanel p)` Removes all the elements from the given panel and restores the default message. |
    | `static void` | `resetTechPanel(javax.swing.JPanel p)` Removes all the elements from the given panel and restores the default message. |
    | `static void` | `setPanel(javax.swing.JPanel parent, javax.swing.JPanel child, javax.swing.JFrame f)` Sets a panel inside a panel and updates the size of the containing window. |

    - ### Methods inherited from class java.lang.Object

      `equals, getClass, hashCode, notify, notifyAll, toString, wait, wait, wait`

- - ### Constructor Detail


    - #### GUIUtils

      ```
      public GUIUtils()
      ```
  - ### Method Detail


    - #### createJPanel

      ```
      public static javax.swing.JPanel createJPanel(java.lang.String name,
                                    java.awt.LayoutManager lm,
                                    java.awt.event.ComponentListener cl)
      ```

      Auxiliar method for creating a named JPanel object.

      Parameters:
      :   `name` - JPanel name.
      :   `lm` - Panel LayoutManager
      :   `cl` - A ComponentListner for handling events

      Returns:
      :   The named JPanel.


    - #### createJPanel

      ```
      public static javax.swing.JPanel createJPanel(java.lang.String name,
                                    java.awt.event.ComponentListener cl)
      ```

      Auxiliar method for creating a named JPanel object.

      Parameters:
      :   `name` - JPanel name.
      :   `cl` - A ComponentListner for handling events

      Returns:
      :   The named JPanel.


    - #### createButton

      ```
      public static java.awt.Button createButton(java.lang.String name,
                                 java.awt.event.ActionListener a)
      ```

      Creates a `Button` with a given label used as name and an
      ActionListener.

      Parameters:
      :   `name` - The name and label for the button.
      :   `a` - An ActionListener for action performing.

      Returns:
      :   The created `Button`.


    - #### createButton

      ```
      public static java.awt.Button createButton(java.lang.String text,
                                 java.lang.String name,
                                 java.awt.event.ActionListener a)
      ```

      Creates a `Button` with a given text, a given name and a given
      ActionListener.

      Parameters:
      :   `text` - The label for the button.
      :   `name` - The name for the button.
      :   `a` - An ActionListener for action performing.

      Returns:
      :   The created `Button`.


    - #### createJButton

      ```
      public static javax.swing.JButton createJButton(java.lang.String text,
                                      java.lang.String name,
                                      java.awt.event.ActionListener a)
      ```

      Creates a `JButton` with a given text, a given name and a given
      ActionListener.

      Parameters:
      :   `text` - The label for the button.
      :   `name` - The name for the button.
      :   `a` - An ActionListener for action performing.

      Returns:
      :   The created `JButton`.


    - #### createJButton

      ```
      public static javax.swing.JButton createJButton(java.lang.String text,
                                      java.awt.event.ActionListener a)
      ```

      Creates a `Button` with a given label used as name and an
      ActionListener.

      Parameters:
      :   `text` - The name and label for the button.
      :   `a` - An ActionListener for action performing.

      Returns:
      :   The created `Button`.


    - #### createChoices

      ```
      public static javax.swing.JComboBox createChoices(java.lang.String name,
                                        java.lang.String[] values,
                                        java.awt.event.ItemListener i)
      ```

      Creates a JComboBox dropdown list.

      Parameters:
      :   `name` - The name for the dropdown list.
      :   `values` - The values to be shown.
      :   `i` - An ItemListener to wait for an action.

      Returns:
      :   The JComboBox list.


    - #### createChoices

      ```
      public static javax.swing.JComboBox createChoices(java.lang.String name,
                                        java.util.ArrayList<java.lang.String> values,
                                        java.awt.event.ItemListener i)
      ```

      Creates a JComboBox dropdown list.

      Parameters:
      :   `name` - The name for the dropdown list.
      :   `values` - The values to be shown.
      :   `i` - An ItemListener to wait for an action.

      Returns:
      :   The JComboBox list.


    - #### createJLabel

      ```
      public static javax.swing.JLabel createJLabel(java.lang.String text,
                                    java.lang.String help)
      ```

      Creates a JLabel with a given text and a given help message

      Parameters:
      :   `text` - Text for the label
      :   `help` - Help text that will appear when user hovers mouse over label

      Returns:
      :   Label


    - #### createJTextField

      ```
      public static javax.swing.JTextField createJTextField(java.lang.String name,
                                            int value,
                                            java.awt.event.FocusListener fl)
      ```

      Creates a new JTextField with the given name, integer value and adds
      a FocusListener.

      Parameters:
      :   `name` - JTextField name.
      :   `value` - JTextField value.
      :   `fl` - FocusListener for this object's events

      Returns:
      :   The newly formed JTextField.


    - #### createJTextField

      ```
      public static javax.swing.JTextField createJTextField(java.lang.String name,
                                            double value,
                                            java.awt.event.FocusListener fl)
      ```

      Creates a new JTextField with the given name, double value and adds
      a FocusListener.

      Parameters:
      :   `name` - JTextField name.
      :   `value` - JTextField value.
      :   `fl` - FocusListener for this object's events

      Returns:
      :   The newly formed JTextField.


    - #### resetTechPanel

      ```
      public static void resetTechPanel(javax.swing.JPanel p)
      ```

      Removes all the elements from the given panel and restores the default
      message.

      Parameters:
      :   `p` - The panel to be reset.


    - #### resetMetricPanel

      ```
      public static void resetMetricPanel(javax.swing.JPanel p)
      ```

      Removes all the elements from the given panel and restores the default
      message.

      Parameters:
      :   `p` - The panel to be reset.


    - #### setPanel

      ```
      public static void setPanel(javax.swing.JPanel parent,
                  javax.swing.JPanel child,
                  javax.swing.JFrame f)
      ```

      Sets a panel inside a panel and updates the size of the containing
      window.

      Parameters:
      :   `parent` - The parent panel.
      :   `child` - The panel to set inside the parent one.
      :   `f` - The frame containing everything.


    - #### getMetricList

      ```
      public static javax.swing.JComboBox getMetricList(ClusteringTechnique t,
                                        ImagePlusHyp ip)
      ```

      Returns a `JComboBox` of `ClusteringMetric` objects to be used
      inside the `ClusteringTechnique` `t`.

      Parameters:
      :   `t` - The `ClusteringTechnique` to host the given
          `ClusteringMetric`.
      :   `ip` - A reference to the working image.

      Returns:
      :   A `JComboBox` object.


- Overview
- Package
- Class
- Use
- Tree
- Deprecated
- Index
- Help

- Prev Class
- Next Class

- Frames
- No Frames

- All Classes

- Summary:
- Nested |
- Field |
- Constr |
- Method

- Detail:
- Field |
- Constr |
- Method
