# Supplementary material for: jClustering, an Open Framework for the Development of 4D Clustering Algorithms
Source: PLoS One. 2013 Aug 22;8(8):e70797. doi: 10.1371/journal.pone.0070797 (PMC3750055; doi:10.1371/journal.pone.0070797)
Supplement: File S1 — Public API for jClustering version 1.2.2. (ZIP) [file pone.0070797.s001.zip › jclustering/ImagePlusHyp.html]

ImagePlusHyp


JavaScript is disabled on your browser.


- Overview
- Package
- Class
- Use
- Tree
- Deprecated
- Index
- Help

- Prev Class
- Next Class

- Frames
- No Frames

- All Classes

- Summary:
- Nested |
- Field |
- Constr |
- Method

- Detail:
- Field |
- Constr |
- Method


jclustering

## Class ImagePlusHyp

- java.lang.Object
- - ij.ImagePlus
  - - jclustering.ImagePlusHyp

- All Implemented Interfaces:
  :   ij.measure.Measurements, java.awt.image.ImageObserver, java.lang.Cloneable, java.lang.Iterable<Voxel>

  ---

    

  ```
  public class ImagePlusHyp
  extends ij.ImagePlus
  implements java.lang.Iterable<Voxel>
  ```

  This class extends `ImagePlus` in order to add a handy `getTAC(int, int, int)`
  method that allows to easily grab time-activity curves.

  Author:
  :   José María Mateos.

- - ### Field Summary

    - ### Fields inherited from class ij.ImagePlus

      `changes, COLOR_256, COLOR_RGB, GRAY16, GRAY32, GRAY8`
    - ### Fields inherited from interface java.awt.image.ImageObserver

      `ABORT, ALLBITS, ERROR, FRAMEBITS, HEIGHT, PROPERTIES, SOMEBITS, WIDTH`
    - ### Fields inherited from interface ij.measure.Measurements

      `ADD_TO_OVERLAY, AREA, AREA_FRACTION, CENTER_OF_MASS, CENTROID, CIRCULARITY, ELLIPSE, FERET, INTEGRATED_DENSITY, INVERT_Y, KURTOSIS, LABELS, LIMIT, MAX_STANDARDS, MEAN, MEDIAN, MIN_MAX, MODE, PERIMETER, RECT, SCIENTIFIC_NOTATION, SHAPE_DESCRIPTORS, SKEWNESS, SLICE, STACK_POSITION, STD_DEV`
  - ### Constructor Summary

    Constructors

    | Constructor and Description |
    | `ImagePlusHyp(ij.ImagePlus ip)` Creates a new ImagePlusHyp using a general ImagePlus. |
  - ### Method Summary

    Methods

    | Modifier and Type | Method and Description |
    | `double[]` | `getTAC(int x, int y, int slice)` Gets the time-activity curve (dixel, after "dynamic pixel") for the given coordinates. |
    | `boolean` | `isNoise(double[] data)` |
    | `java.util.Iterator<Voxel>` | `iterator()` |

    - ### Methods inherited from class ij.ImagePlus

      `addImageListener, clone, close, convertIndexToPosition, copy, copyScale, createEmptyStack, createHyperStack, createImagePlus, createLut, createNewRoi, deleteRoi, draw, draw, duplicate, flatten, flush, getBitDepth, getBufferedImage, getBytesPerPixel, getC, getCalibration, getCanvas, getChannel, getChannelProcessor, getClipboard, getCurrentSlice, getDefault16bitRange, getDimensions, getDimensions, getDisplayRangeMax, getDisplayRangeMin, getFileInfo, getFrame, getGlobalCalibration, getHeight, getHideOverlay, getID, getImage, getImageStack, getImageStackSize, getLocalCalibration, getLocationAsString, getLuts, getMask, getNChannels, getNDimensions, getNFrames, getNSlices, getOpenAsHyperStack, getOriginalFileInfo, getOverlay, getPixel, getProcessor, getProperties, getProperty, getRoi, getShortTitle, getSlice, getStack, getStackIndex, getStackSize, getStartTime, getStatistics, getStatistics, getStatistics, getStatistics, getT, getTitle, getType, getWidth, getWindow, getZ, hide, imageUpdate, isComposite, isDisplayedHyperStack, isHyperStack, isInvertedLut, isLocked, isProcessor, isVisible, killRoi, killStack, lock, lockSilently, mouseMoved, paste, removeImageListener, repaintWindow, resetClipboard, resetDisplayRange, resetStack, restoreRoi, revert, saveRoi, setActivated, setC, setCalibration, setColor, setDefault16bitRange, setDimensions, setDisplayRange, setDisplayRange, setFileInfo, setGlobalCalibration, setHideOverlay, setIgnoreFlush, setImage, setImage, setOpenAsHyperStack, setOverlay, setOverlay, setOverlay, setPosition, setPosition, setPositionWithoutUpdate, setProcessor, setProcessor, setProperty, setRoi, setRoi, setRoi, setRoi, setSlice, setSliceWithoutUpdate, setStack, setStack, setStack, setT, setTitle, setWindow, setZ, show, show, startTiming, toString, trimProcessor, unlock, updateAndDraw, updateAndRepaintWindow, updateChannelAndDraw, updateImage, updatePosition, updateStatusbarValue`
    - ### Methods inherited from class java.lang.Object

      `equals, getClass, hashCode, notify, notifyAll, wait, wait, wait`

- - ### Constructor Detail


    - #### ImagePlusHyp

      ```
      public ImagePlusHyp(ij.ImagePlus ip)
      ```

      Creates a new ImagePlusHyp using a general ImagePlus.

      Parameters:
      :   `ip` - The ImagePlus used to construct this class.
  - ### Method Detail


    - #### getTAC

      ```
      public double[] getTAC(int x,
                    int y,
                    int slice)
      ```

      Gets the time-activity curve (dixel, after "dynamic pixel") for the
      given coordinates. Please note that this method returns the calibrated
      curve according to calibration data present in the image header, not
      the raw values. Please refer to ImageJ's `Calibration` object
      documentation for more information regarding this.

      Parameters:
      :   `x` - The x coordinate of the desired dixel.
      :   `y` - The y coordinate of the desired dixel.
      :   `slice` - Slice (1-based) of the desired dixel.

      Returns:
      :   A double array containing the values for the given voxel on each
          frame, or null if the coordinates are not valid.


    - #### isNoise

      ```
      public boolean isNoise(double[] data)
      ```

      Parameters:
      :   `data` - The TAC to be tested

      Returns:
      :   true if the given TAC is noise with respect to this image. A
          TAC is considered noise if the absolute value of its minimum value is
          greater or equal than its maximum value. This is a very simplistic
          approach, but works in most cases.


    - #### iterator

      ```
      public java.util.Iterator<Voxel> iterator()
      ```

      **Specified by:**
      :   `iterator` in interface `java.lang.Iterable<Voxel>`


- Overview
- Package
- Class
- Use
- Tree
- Deprecated
- Index
- Help

- Prev Class
- Next Class

- Frames
- No Frames

- All Classes

- Summary:
- Nested |
- Field |
- Constr |
- Method

- Detail:
- Field |
- Constr |
- Method
