# Supplementary material for: jClustering, an Open Framework for the Development of 4D Clustering Algorithms
Source: PLoS One. 2013 Aug 22;8(8):e70797. doi: 10.1371/journal.pone.0070797 (PMC3750055; doi:10.1371/journal.pone.0070797)
Supplement: File S1 — Public API for jClustering version 1.2.2. (ZIP) [file pone.0070797.s001.zip › jclustering/ImagePlusHypIterator.html]

ImagePlusHypIterator


JavaScript is disabled on your browser.


- Overview
- Package
- Class
- Use
- Tree
- Deprecated
- Index
- Help

- Prev Class
- Next Class

- Frames
- No Frames

- All Classes

- Summary:
- Nested |
- Field |
- Constr |
- Method

- Detail:
- Field |
- Constr |
- Method


jclustering

## Class ImagePlusHypIterator

- java.lang.Object
- - jclustering.ImagePlusHypIterator

- All Implemented Interfaces:
  :   java.util.Iterator<Voxel>

  ---

    

  ```
  public class ImagePlusHypIterator
  extends java.lang.Object
  implements java.util.Iterator<Voxel>
  ```

  Provides an `Iterator` for an `ImagePlusHyp` object.

  Author:
  :   José María Mateos.

- - ### Constructor Summary

    Constructors

    | Constructor and Description |
    | `ImagePlusHypIterator(ImagePlusHyp ip)` Public constructor. |
  - ### Method Summary

    Methods

    | Modifier and Type | Method and Description |
    | `boolean` | `hasNext()` |
    | `Voxel` | `next()` |
    | `void` | `remove()` |

    - ### Methods inherited from class java.lang.Object

      `equals, getClass, hashCode, notify, notifyAll, toString, wait, wait, wait`

- - ### Constructor Detail


    - #### ImagePlusHypIterator

      ```
      public ImagePlusHypIterator(ImagePlusHyp ip)
      ```

      Public constructor.

      Parameters:
      :   `ip` - The `ImagePlusHyp` object to be iterated.
  - ### Method Detail


    - #### hasNext

      ```
      public boolean hasNext()
      ```

      **Specified by:**
      :   `hasNext` in interface `java.util.Iterator<Voxel>`


    - #### next

      ```
      public Voxel next()
      ```

      **Specified by:**
      :   `next` in interface `java.util.Iterator<Voxel>`


    - #### remove

      ```
      public void remove()
                  throws java.lang.UnsupportedOperationException
      ```

      **Specified by:**
      :   `remove` in interface `java.util.Iterator<Voxel>`

      Throws:
      :   `java.lang.UnsupportedOperationException`


- Overview
- Package
- Class
- Use
- Tree
- Deprecated
- Index
- Help

- Prev Class
- Next Class

- Frames
- No Frames

- All Classes

- Summary:
- Nested |
- Field |
- Constr |
- Method

- Detail:
- Field |
- Constr |
- Method
