# Supplementary material for: jClustering, an Open Framework for the Development of 4D Clustering Algorithms
Source: PLoS One. 2013 Aug 22;8(8):e70797. doi: 10.1371/journal.pone.0070797 (PMC3750055; doi:10.1371/journal.pone.0070797)
Supplement: File S1 — Public API for jClustering version 1.2.2. (ZIP) [file pone.0070797.s001.zip › jclustering/JClustering_.html]

JClustering\_


JavaScript is disabled on your browser.


- Overview
- Package
- Class
- Use
- Tree
- Deprecated
- Index
- Help

- Prev Class
- Next Class

- Frames
- No Frames

- All Classes

- Summary:
- Nested |
- Field |
- Constr |
- Method

- Detail:
- Field |
- Constr |
- Method


jclustering

## Class JClustering\_

- java.lang.Object
- - jclustering.JClustering\_

- All Implemented Interfaces:
  :   ij.plugin.filter.PlugInFilter, java.awt.event.ActionListener, java.awt.event.ComponentListener, java.awt.event.ItemListener, java.util.EventListener

  ---

    

  ```
  public class JClustering_
  extends java.lang.Object
  implements ij.plugin.filter.PlugInFilter, java.awt.event.ActionListener, java.awt.event.ItemListener, java.awt.event.ComponentListener
  ```

  JClustering ImageJ Plugin.

  This plugin is intended to be used as a development framework for developing
  clustering algorithms to be used in nuclear medicine dynamic studies or
  any other modality of dynamic imaging.
  Examples of said clustering algorithms are k-means, leader-follower,
  Principal Component Analysis, Independent Component Analysis, Factorial
  Analysis...

  Author:
  :   José María Mateos.

- - ### Field Summary

    - ### Fields inherited from interface ij.plugin.filter.PlugInFilter

      `CONVERT_TO_FLOAT, DOES_16, DOES_32, DOES_8C, DOES_8G, DOES_ALL, DOES_RGB, DOES_STACKS, DONE, FINAL_PROCESSING, KEEP_THRESHOLD, NO_CHANGES, NO_IMAGE_REQUIRED, NO_UNDO, PARALLELIZE_IMAGES, PARALLELIZE_STACKS, ROI_REQUIRED, SNAPSHOT, STACK_REQUIRED, SUPPORTS_MASKING`
  - ### Constructor Summary

    Constructors

    | Constructor and Description |
    | `JClustering_()` |
  - ### Method Summary

    Methods

    | Modifier and Type | Method and Description |
    | `void` | `actionPerformed(java.awt.event.ActionEvent e)` |
    | `void` | `componentHidden(java.awt.event.ComponentEvent arg0)` |
    | `void` | `componentMoved(java.awt.event.ComponentEvent arg0)` |
    | `void` | `componentResized(java.awt.event.ComponentEvent arg0)` |
    | `void` | `componentShown(java.awt.event.ComponentEvent arg0)` |
    | `void` | `itemStateChanged(java.awt.event.ItemEvent arg0)` |
    | `static void` | `main(java.lang.String[] args)` Main method for testing purposes. |
    | `void` | `run(ij.process.ImageProcessor ip)` |
    | `int` | `setup(java.lang.String arg, ij.ImagePlus imp)` |

    - ### Methods inherited from class java.lang.Object

      `equals, getClass, hashCode, notify, notifyAll, toString, wait, wait, wait`

- - ### Constructor Detail


    - #### JClustering\_

      ```
      public JClustering_()
      ```
  - ### Method Detail


    - #### run

      ```
      public void run(ij.process.ImageProcessor ip)
      ```

      **Specified by:**
      :   `run` in interface `ij.plugin.filter.PlugInFilter`


    - #### setup

      ```
      public int setup(java.lang.String arg,
              ij.ImagePlus imp)
      ```

      **Specified by:**
      :   `setup` in interface `ij.plugin.filter.PlugInFilter`


    - #### actionPerformed

      ```
      public void actionPerformed(java.awt.event.ActionEvent e)
      ```

      **Specified by:**
      :   `actionPerformed` in interface `java.awt.event.ActionListener`


    - #### itemStateChanged

      ```
      public void itemStateChanged(java.awt.event.ItemEvent arg0)
      ```

      **Specified by:**
      :   `itemStateChanged` in interface `java.awt.event.ItemListener`


    - #### componentShown

      ```
      public void componentShown(java.awt.event.ComponentEvent arg0)
      ```

      **Specified by:**
      :   `componentShown` in interface `java.awt.event.ComponentListener`


    - #### componentHidden

      ```
      public void componentHidden(java.awt.event.ComponentEvent arg0)
      ```

      **Specified by:**
      :   `componentHidden` in interface `java.awt.event.ComponentListener`


    - #### componentMoved

      ```
      public void componentMoved(java.awt.event.ComponentEvent arg0)
      ```

      **Specified by:**
      :   `componentMoved` in interface `java.awt.event.ComponentListener`


    - #### componentResized

      ```
      public void componentResized(java.awt.event.ComponentEvent arg0)
      ```

      **Specified by:**
      :   `componentResized` in interface `java.awt.event.ComponentListener`


    - #### main

      ```
      public static void main(java.lang.String[] args)
      ```

      Main method for testing purposes.

      Parameters:
      :   `args` - Input arguments from the command line, if any.


- Overview
- Package
- Class
- Use
- Tree
- Deprecated
- Index
- Help

- Prev Class
- Next Class

- Frames
- No Frames

- All Classes

- Summary:
- Nested |
- Field |
- Constr |
- Method

- Detail:
- Field |
- Constr |
- Method
