# Supplementary material for: jClustering, an Open Framework for the Development of 4D Clustering Algorithms
Source: PLoS One. 2013 Aug 22;8(8):e70797. doi: 10.1371/journal.pone.0070797 (PMC3750055; doi:10.1371/journal.pone.0070797)
Supplement: File S1 — Public API for jClustering version 1.2.2. (ZIP) [file pone.0070797.s001.zip › jclustering/MathUtils.html]

MathUtils


JavaScript is disabled on your browser.


- Overview
- Package
- Class
- Use
- Tree
- Deprecated
- Index
- Help

- Prev Class
- Next Class

- Frames
- No Frames

- All Classes

- Summary:
- Nested |
- Field |
- Constr |
- Method

- Detail:
- Field |
- Constr |
- Method


jclustering

## Class MathUtils

- java.lang.Object
- - jclustering.MathUtils

- ---

    

  ```
  public class MathUtils
  extends java.lang.Object
  ```

  Math helper class.

  Author:
  :   José María Mateos.

- - ### Constructor Summary

    Constructors

    | Constructor and Description |
    | `MathUtils()` |
  - ### Method Summary

    Methods

    | Modifier and Type | Method and Description |
    | `static int` | `getMaxIndex(double[] d)` Returns the index for the maximum value of the array. |
    | `static double` | `nrmsd(double[] x1, double[] x2)` Computes the normalized RMSD for the given TACs |
    | `static double` | `rmsd(double[] x1, double[] x2)` Computes the root-mean-square deviation for the given TACs |
    | `static double[]` | `smooth(double[] data)` Smooths the given TAC using a 5-point filtering. |

    - ### Methods inherited from class java.lang.Object

      `equals, getClass, hashCode, notify, notifyAll, toString, wait, wait, wait`

- - ### Constructor Detail


    - #### MathUtils

      ```
      public MathUtils()
      ```
  - ### Method Detail


    - #### getMaxIndex

      ```
      public static int getMaxIndex(double[] d)
      ```

      Returns the index for the maximum value of the array.

      Parameters:
      :   `d` - A double array.

      Returns:
      :   The index for the maximum value.


    - #### smooth

      ```
      public static double[] smooth(double[] data)
      ```

      Smooths the given TAC using a 5-point filtering.

      Parameters:
      :   `data` - The raw TAC.

      Returns:
      :   The smoothed TAC.


    - #### rmsd

      ```
      public static double rmsd(double[] x1,
                double[] x2)
      ```

      Computes the root-mean-square deviation for the given TACs

      Parameters:
      :   `x1` - TAC 1
      :   `x2` - TAC 2

      Returns:
      :   RMSD for the given TACs


    - #### nrmsd

      ```
      public static double nrmsd(double[] x1,
                 double[] x2)
      ```

      Computes the normalized RMSD for the given TACs

      Parameters:
      :   `x1` - TAC 1
      :   `x2` - TAC 2

      Returns:
      :   NRMSD for the given TACs


- Overview
- Package
- Class
- Use
- Tree
- Deprecated
- Index
- Help

- Prev Class
- Next Class

- Frames
- No Frames

- All Classes

- Summary:
- Nested |
- Field |
- Constr |
- Method

- Detail:
- Field |
- Constr |
- Method
