# Supplementary material for: jClustering, an Open Framework for the Development of 4D Clustering Algorithms
Source: PLoS One. 2013 Aug 22;8(8):e70797. doi: 10.1371/journal.pone.0070797 (PMC3750055; doi:10.1371/journal.pone.0070797)
Supplement: File S1 — Public API for jClustering version 1.2.2. (ZIP) [file pone.0070797.s001.zip › jclustering/metrics/class-use/ClusteringMetric.html]

Uses of Class jclustering.metrics.ClusteringMetric


JavaScript is disabled on your browser.


- Overview
- Package
- Class
- Use
- Tree
- Deprecated
- Index
- Help

- Prev
- Next

- Frames
- No Frames

- All Classes

## Uses of Class jclustering.metrics.ClusteringMetric

- Packages that use ClusteringMetric

  | Package | Description |
  |  |  |
  | --- | --- |
  | jclustering |  |
  | jclustering.metrics |  |
  | jclustering.techniques |  |
- - ### Uses of ClusteringMetric in jclustering

    Methods in jclustering that return ClusteringMetric

    | Modifier and Type | Method and Description |
    |  |  |
    | --- | --- |
    | `static ClusteringMetric` | Utils.`getClusteringMetric(java.lang.String name, ImagePlusHyp ip)` Builds a new instance for a `ClusteringMetric` object and returns it. |
  - ### Uses of ClusteringMetric in jclustering.metrics

    Subclasses of ClusteringMetric in jclustering.metrics

    | Modifier and Type | Class and Description |
    |  |  |
    | --- | --- |
    | `class` | `Correlation` Classical correlation score between two given TACs (data type `double[]`). |
    | `class` | `Mahalanobis` Implements a Mahanalnobis distance. |
    | `class` | `PNorm` This `ClusteringMetric` implements a p-norm distance. |
    | `class` | `RMSD` Root-mean-square deviation between two given TACs (data type `double[]`). |
  - ### Uses of ClusteringMetric in jclustering.techniques

    Methods in jclustering.techniques that return ClusteringMetric

    | Modifier and Type | Method and Description |
    |  |  |
    | --- | --- |
    | `ClusteringMetric` | ClusteringTechnique.`getMetric()` |

    Methods in jclustering.techniques with parameters of type ClusteringMetric

    | Modifier and Type | Method and Description |
    |  |  |
    | --- | --- |
    | `void` | ClusteringTechnique.`setMetric(ClusteringMetric m)` Sets the current ClusteringMetric |

- Overview
- Package
- Class
- Use
- Tree
- Deprecated
- Index
- Help

- Prev
- Next

- Frames
- No Frames

- All Classes
