# Supplementary material for: jClustering, an Open Framework for the Development of 4D Clustering Algorithms
Source: PLoS One. 2013 Aug 22;8(8):e70797. doi: 10.1371/journal.pone.0070797 (PMC3750055; doi:10.1371/journal.pone.0070797)
Supplement: File S1 — Public API for jClustering version 1.2.2. (ZIP) [file pone.0070797.s001.zip › jclustering/metrics/ClusteringMetric.html]

ClusteringMetric


JavaScript is disabled on your browser.


- Overview
- Package
- Class
- Use
- Tree
- Deprecated
- Index
- Help

- Prev Class
- Next Class

- Frames
- No Frames

- All Classes

- Summary:
- Nested |
- Field |
- Constr |
- Method

- Detail:
- Field |
- Constr |
- Method


jclustering.metrics

## Class ClusteringMetric

- java.lang.Object
- - jclustering.metrics.ClusteringMetric

- All Implemented Interfaces:
  :   java.awt.event.ActionListener, java.awt.event.ItemListener, java.util.EventListener

  Direct Known Subclasses:
  :   Correlation, Mahalanobis, PNorm, RMSD

  ---

    

  ```
  public abstract class ClusteringMetric
  extends java.lang.Object
  implements java.awt.event.ActionListener, java.awt.event.ItemListener
  ```

  This abstract class provides a template with the basic functions that a
  metric should implement, specially the distance(double [], double[]) method.

  Author:
  :   José María Mateos.

- - ### Constructor Summary

    Constructors

    | Constructor and Description |
    | `ClusteringMetric()` |
  - ### Method Summary

    Methods

    | Modifier and Type | Method and Description |
    | `void` | `actionPerformed(java.awt.event.ActionEvent e)` |
    | `abstract double` | `distance(double[] data, double[] centroid)` Computes the distance between to TACs according to this particular metric. |
    | `double` | `distance(Voxel v, double[] centroid)` Provides a shortcut for computing the distance between a `Voxel` and any TAC. |
    | `javax.swing.JPanel` | `getConfig()` Builds a configuration `Panel` that will provide all the necessary interfaces for the technique configuration. |
    | `java.lang.String` | `getName()` |
    | `void` | `init()` If the metric needs some previous computations, it should override this method. |
    | `boolean` | `isNoise(double[] tac)` Ease of access for the `ImagePlusHyp.isNoise(double[])` method. |
    | `boolean` | `isNoise(Voxel v)` Ease of access for the `ImagePlusHyp.isNoise(double[])` method. |
    | `void` | `itemStateChanged(java.awt.event.ItemEvent e)` |
    | `javax.swing.JPanel` | `makeConfig()` This function is called only once and returns the configuration panel that will be called by `getConfig()` on each successive call. |
    | `void` | `setup(ImagePlusHyp ip)` Setup method, as the constructor will always be called empty. |
    | `void` | `skip_noisy(boolean skip_noisy)` Sets the skip\_noisy variable for this metric. |

    - ### Methods inherited from class java.lang.Object

      `equals, getClass, hashCode, notify, notifyAll, toString, wait, wait, wait`

- - ### Constructor Detail


    - #### ClusteringMetric

      ```
      public ClusteringMetric()
      ```
  - ### Method Detail


    - #### distance

      ```
      public abstract double distance(double[] data,
                    double[] centroid)
      ```

      Computes the distance between to TACs according to this particular
      metric. Extending classes must implement this method.

      Parameters:
      :   `data` - The TAC to compare.
      :   `centroid` - The cluster centroid.

      Returns:
      :   The distance between both arrays.


    - #### distance

      ```
      public double distance(Voxel v,
                    double[] centroid)
      ```

      Provides a shortcut for computing the distance between a `Voxel`
      and any TAC.

      Parameters:
      :   `v` - The Voxel which distance is to be computed.
      :   `centroid` - The cluster centroid.

      Returns:
      :   The distance between both TACs.


    - #### getName

      ```
      public java.lang.String getName()
      ```

      Returns:
      :   The name of this metric.


    - #### getConfig

      ```
      public javax.swing.JPanel getConfig()
      ```

      Builds a configuration `Panel` that will provide all the necessary
      interfaces for the technique configuration. If implemented, the technique
      class must also implement the necessary listeners.

      Developers may use the classes provided in the `GUIUtils` static
      methods.

      Returns:
      :   `null` by default, or the appropriate `Panel` if
          implemented.


    - #### makeConfig

      ```
      public javax.swing.JPanel makeConfig()
      ```

      This function is called only once and returns the configuration panel
      that will be called by `getConfig()` on each successive call.
      Needs to be overridden by the extending classes.

      Returns:
      :   The configuration panel returned by `getConfig()`.


    - #### setup

      ```
      public void setup(ImagePlusHyp ip)
      ```

      Setup method, as the constructor will always be called empty. Provides a
      reference to the working image.

      Parameters:
      :   `ip` - The working image.


    - #### isNoise

      ```
      public boolean isNoise(double[] tac)
      ```

      Ease of access for the `ImagePlusHyp.isNoise(double[])` method.

      Parameters:
      :   `tac` - The TAC to be tested.

      Returns:
      :   true if it noise.


    - #### isNoise

      ```
      public boolean isNoise(Voxel v)
      ```

      Ease of access for the `ImagePlusHyp.isNoise(double[])` method.

      Parameters:
      :   `v` - The voxel to be tested.

      Returns:
      :   true if it noise.


    - #### init

      ```
      public void init()
      ```

      If the metric needs some previous computations, it should override
      this method. This is called just once prior to
      `ClusteringTechnique.process()`. By default it does nothing.


    - #### skip\_noisy

      ```
      public void skip_noisy(boolean skip_noisy)
      ```

      Sets the skip\_noisy variable for this metric.

      Parameters:
      :   `skip_noisy` - Tells the init process whether to skip noisy voxels.


    - #### actionPerformed

      ```
      public void actionPerformed(java.awt.event.ActionEvent e)
      ```

      **Specified by:**
      :   `actionPerformed` in interface `java.awt.event.ActionListener`


    - #### itemStateChanged

      ```
      public void itemStateChanged(java.awt.event.ItemEvent e)
      ```

      **Specified by:**
      :   `itemStateChanged` in interface `java.awt.event.ItemListener`


- Overview
- Package
- Class
- Use
- Tree
- Deprecated
- Index
- Help

- Prev Class
- Next Class

- Frames
- No Frames

- All Classes

- Summary:
- Nested |
- Field |
- Constr |
- Method

- Detail:
- Field |
- Constr |
- Method
