# Supplementary material for: jClustering, an Open Framework for the Development of 4D Clustering Algorithms
Source: PLoS One. 2013 Aug 22;8(8):e70797. doi: 10.1371/journal.pone.0070797 (PMC3750055; doi:10.1371/journal.pone.0070797)
Supplement: File S1 — Public API for jClustering version 1.2.2. (ZIP) [file pone.0070797.s001.zip › jclustering/metrics/Correlation.html]

Correlation


JavaScript is disabled on your browser.


- Overview
- Package
- Class
- Use
- Tree
- Deprecated
- Index
- Help

- Prev Class
- Next Class

- Frames
- No Frames

- All Classes

- Summary:
- Nested |
- Field |
- Constr |
- Method

- Detail:
- Field |
- Constr |
- Method


jclustering.metrics

## Class Correlation

- java.lang.Object
- - jclustering.metrics.ClusteringMetric
  - - jclustering.metrics.Correlation

- All Implemented Interfaces:
  :   java.awt.event.ActionListener, java.awt.event.ItemListener, java.util.EventListener

  ---

    

  ```
  public class Correlation
  extends ClusteringMetric
  ```

  Classical correlation score between two given TACs (data type
  `double[]`).

  Author:
  :   José María Mateos.

- - ### Constructor Summary

    Constructors

    | Constructor and Description |
    | `Correlation()` |
  - ### Method Summary

    Methods

    | Modifier and Type | Method and Description |
    | `double` | `distance(double[] centroid, double[] data)` Computes the distance between to TACs according to this particular metric. |
    | `void` | `init()` If the metric needs some previous computations, it should override this method. |

    - ### Methods inherited from class jclustering.metrics.ClusteringMetric

      `actionPerformed, distance, getConfig, getName, isNoise, isNoise, itemStateChanged, makeConfig, setup, skip_noisy`
    - ### Methods inherited from class java.lang.Object

      `equals, getClass, hashCode, notify, notifyAll, toString, wait, wait, wait`

- - ### Constructor Detail


    - #### Correlation

      ```
      public Correlation()
      ```
  - ### Method Detail


    - #### distance

      ```
      public double distance(double[] centroid,
                    double[] data)
      ```

      **Description copied from class: `ClusteringMetric`**

      Computes the distance between to TACs according to this particular
      metric. Extending classes must implement this method.

      **Specified by:**
      :   `distance` in class `ClusteringMetric`

      Parameters:
      :   `centroid` - The TAC to compare.
      :   `data` - The cluster centroid.

      Returns:
      :   The distance between both arrays.


    - #### init

      ```
      public void init()
      ```

      **Description copied from class: `ClusteringMetric`**

      If the metric needs some previous computations, it should override
      this method. This is called just once prior to
      `ClusteringTechnique.process()`. By default it does nothing.

      **Overrides:**
      :   `init` in class `ClusteringMetric`


- Overview
- Package
- Class
- Use
- Tree
- Deprecated
- Index
- Help

- Prev Class
- Next Class

- Frames
- No Frames

- All Classes

- Summary:
- Nested |
- Field |
- Constr |
- Method

- Detail:
- Field |
- Constr |
- Method
