# Supplementary material for: jClustering, an Open Framework for the Development of 4D Clustering Algorithms
Source: PLoS One. 2013 Aug 22;8(8):e70797. doi: 10.1371/journal.pone.0070797 (PMC3750055; doi:10.1371/journal.pone.0070797)
Supplement: File S1 — Public API for jClustering version 1.2.2. (ZIP) [file pone.0070797.s001.zip › jclustering/metrics/package-frame.html]

jclustering.metrics


# jclustering.metrics

## Classes

- ClusteringMetric
- Correlation
- Mahalanobis
- PNorm
- RMSD
