# Supplementary material for: jClustering, an Open Framework for the Development of 4D Clustering Algorithms
Source: PLoS One. 2013 Aug 22;8(8):e70797. doi: 10.1371/journal.pone.0070797 (PMC3750055; doi:10.1371/journal.pone.0070797)
Supplement: File S1 — Public API for jClustering version 1.2.2. (ZIP) [file pone.0070797.s001.zip › jclustering/metrics/package-summary.html]

jclustering.metrics


JavaScript is disabled on your browser.


- Overview
- Package
- Class
- Use
- Tree
- Deprecated
- Index
- Help

- Prev Package
- Next Package

- Frames
- No Frames

- All Classes

# Package jclustering.metrics

- Class Summary

  | Class | Description |
  |  |  |
  | --- | --- |
  | ClusteringMetric | This abstract class provides a template with the basic functions that a metric should implement, specially the distance(double [], double[]) method. |
  | Correlation | Classical correlation score between two given TACs (data type `double[]`). |
  | Mahalanobis | Implements a Mahanalnobis distance. |
  | PNorm | This `ClusteringMetric` implements a p-norm distance. |
  | RMSD | Root-mean-square deviation between two given TACs (data type `double[]`). |

- Overview
- Package
- Class
- Use
- Tree
- Deprecated
- Index
- Help

- Prev Package
- Next Package

- Frames
- No Frames

- All Classes
