# Supplementary material for: jClustering, an Open Framework for the Development of 4D Clustering Algorithms
Source: PLoS One. 2013 Aug 22;8(8):e70797. doi: 10.1371/journal.pone.0070797 (PMC3750055; doi:10.1371/journal.pone.0070797)
Supplement: File S1 — Public API for jClustering version 1.2.2. (ZIP) [file pone.0070797.s001.zip › jclustering/metrics/package-tree.html]

jclustering.metrics Class Hierarchy


JavaScript is disabled on your browser.


- Overview
- Package
- Class
- Use
- Tree
- Deprecated
- Index
- Help

- Prev
- Next

- Frames
- No Frames

- All Classes

# Hierarchy For Package jclustering.metrics

Package Hierarchies:

- All Packages

## Class Hierarchy

- java.lang.Object
  - jclustering.metrics.ClusteringMetric (implements java.awt.event.ActionListener, java.awt.event.ItemListener)
    - jclustering.metrics.Correlation
    - jclustering.metrics.Mahalanobis
    - jclustering.metrics.PNorm (implements java.awt.event.FocusListener)
    - jclustering.metrics.RMSD

- Overview
- Package
- Class
- Use
- Tree
- Deprecated
- Index
- Help

- Prev
- Next

- Frames
- No Frames

- All Classes
