# Supplementary material for: jClustering, an Open Framework for the Development of 4D Clustering Algorithms
Source: PLoS One. 2013 Aug 22;8(8):e70797. doi: 10.1371/journal.pone.0070797 (PMC3750055; doi:10.1371/journal.pone.0070797)
Supplement: File S1 — Public API for jClustering version 1.2.2. (ZIP) [file pone.0070797.s001.zip › jclustering/metrics/PNorm.html]

PNorm


JavaScript is disabled on your browser.


- Overview
- Package
- Class
- Use
- Tree
- Deprecated
- Index
- Help

- Prev Class
- Next Class

- Frames
- No Frames

- All Classes

- Summary:
- Nested |
- Field |
- Constr |
- Method

- Detail:
- Field |
- Constr |
- Method


jclustering.metrics

## Class PNorm

- java.lang.Object
- - jclustering.metrics.ClusteringMetric
  - - jclustering.metrics.PNorm

- All Implemented Interfaces:
  :   java.awt.event.ActionListener, java.awt.event.FocusListener, java.awt.event.ItemListener, java.util.EventListener

  ---

    

  ```
  public class PNorm
  extends ClusteringMetric
  implements java.awt.event.FocusListener
  ```

  This `ClusteringMetric` implements a p-norm
  distance.

  The only variable that this metric needs to set is `p`, which has a
  default value of `2.0`. In this case, the distance is an Euclidean
  distance.

  Author:
  :   José María Mateos.

- - ### Constructor Summary

    Constructors

    | Constructor and Description |
    | `PNorm()` |
  - ### Method Summary

    Methods

    | Modifier and Type | Method and Description |
    | `double` | `distance(double[] centroid, double[] data)` Computes the distance between to TACs according to this particular metric. |
    | `void` | `focusGained(java.awt.event.FocusEvent e)` |
    | `void` | `focusLost(java.awt.event.FocusEvent e)` |
    | `javax.swing.JPanel` | `makeConfig()` This function is called only once and returns the configuration panel that will be called by `ClusteringMetric.getConfig()` on each successive call. |

    - ### Methods inherited from class jclustering.metrics.ClusteringMetric

      `actionPerformed, distance, getConfig, getName, init, isNoise, isNoise, itemStateChanged, setup, skip_noisy`
    - ### Methods inherited from class java.lang.Object

      `equals, getClass, hashCode, notify, notifyAll, toString, wait, wait, wait`

- - ### Constructor Detail


    - #### PNorm

      ```
      public PNorm()
      ```
  - ### Method Detail


    - #### distance

      ```
      public double distance(double[] centroid,
                    double[] data)
      ```

      **Description copied from class: `ClusteringMetric`**

      Computes the distance between to TACs according to this particular
      metric. Extending classes must implement this method.

      **Specified by:**
      :   `distance` in class `ClusteringMetric`

      Parameters:
      :   `centroid` - The TAC to compare.
      :   `data` - The cluster centroid.

      Returns:
      :   The distance between both arrays.


    - #### makeConfig

      ```
      public javax.swing.JPanel makeConfig()
      ```

      **Description copied from class: `ClusteringMetric`**

      This function is called only once and returns the configuration panel
      that will be called by `ClusteringMetric.getConfig()` on each successive call.
      Needs to be overridden by the extending classes.

      **Overrides:**
      :   `makeConfig` in class `ClusteringMetric`

      Returns:
      :   The configuration panel returned by `ClusteringMetric.getConfig()`.


    - #### focusLost

      ```
      public void focusLost(java.awt.event.FocusEvent e)
      ```

      **Specified by:**
      :   `focusLost` in interface `java.awt.event.FocusListener`


    - #### focusGained

      ```
      public void focusGained(java.awt.event.FocusEvent e)
      ```

      **Specified by:**
      :   `focusGained` in interface `java.awt.event.FocusListener`


- Overview
- Package
- Class
- Use
- Tree
- Deprecated
- Index
- Help

- Prev Class
- Next Class

- Frames
- No Frames

- All Classes

- Summary:
- Nested |
- Field |
- Constr |
- Method

- Detail:
- Field |
- Constr |
- Method
