# Supplementary material for: jClustering, an Open Framework for the Development of 4D Clustering Algorithms
Source: PLoS One. 2013 Aug 22;8(8):e70797. doi: 10.1371/journal.pone.0070797 (PMC3750055; doi:10.1371/journal.pone.0070797)
Supplement: File S1 — Public API for jClustering version 1.2.2. (ZIP) [file pone.0070797.s001.zip › jclustering/package-frame.html]

jclustering


# jclustering

## Classes

- Cluster
- Constants
- FileSaver
- GUIUtils
- ImagePlusHyp
- ImagePlusHypIterator
- JClustering\_
- MathUtils
- TimeVectorReader
- Utils
- Voxel
