# Supplementary material for: jClustering, an Open Framework for the Development of 4D Clustering Algorithms
Source: PLoS One. 2013 Aug 22;8(8):e70797. doi: 10.1371/journal.pone.0070797 (PMC3750055; doi:10.1371/journal.pone.0070797)
Supplement: File S1 — Public API for jClustering version 1.2.2. (ZIP) [file pone.0070797.s001.zip › jclustering/package-summary.html]

jclustering


JavaScript is disabled on your browser.


- Overview
- Package
- Class
- Use
- Tree
- Deprecated
- Index
- Help

- Prev Package
- Next Package

- Frames
- No Frames

- All Classes

# Package jclustering

- Class Summary

  | Class | Description |
  |  |  |
  | --- | --- |
  | Cluster | Implements a cluster class. |
  | Constants | Define the necessary constants. |
  | FileSaver | Class for file saving. |
  | GUIUtils | Auxiliar class with static utility methods for GUI creation |
  | ImagePlusHyp | This class extends `ImagePlus` in order to add a handy `ImagePlusHyp.getTAC(int, int, int)` method that allows to easily grab time-activity curves. |
  | ImagePlusHypIterator | Provides an `Iterator` for an `ImagePlusHyp` object. |
  | JClustering\_ | JClustering ImageJ Plugin. |
  | MathUtils | Math helper class. |
  | TimeVectorReader | This class reads data from a text file with two columns separated by a space: each row contains the starting time (first column) and ending time (second column). |
  | Utils | Auxiliary class with misc static methods |
  | Voxel | Simple data transfer object to ease the analysis of all the TACs in a given image. |

- Overview
- Package
- Class
- Use
- Tree
- Deprecated
- Index
- Help

- Prev Package
- Next Package

- Frames
- No Frames

- All Classes
