# Supplementary material for: jClustering, an Open Framework for the Development of 4D Clustering Algorithms
Source: PLoS One. 2013 Aug 22;8(8):e70797. doi: 10.1371/journal.pone.0070797 (PMC3750055; doi:10.1371/journal.pone.0070797)
Supplement: File S1 — Public API for jClustering version 1.2.2. (ZIP) [file pone.0070797.s001.zip › jclustering/package-use.html]

Uses of Package jclustering


JavaScript is disabled on your browser.


- Overview
- Package
- Class
- Use
- Tree
- Deprecated
- Index
- Help

- Prev
- Next

- Frames
- No Frames

- All Classes

# Uses of Package jclustering

- Packages that use jclustering

  | Package | Description |
  |  |  |
  | --- | --- |
  | jclustering |  |
  | jclustering.metrics |  |
  | jclustering.techniques |  |
- Classes in jclustering used by jclustering

  | Class and Description |
  |  |
  | --- |
  | Cluster Implements a cluster class. |
  | ImagePlusHyp This class extends `ImagePlus` in order to add a handy `ImagePlusHyp.getTAC(int, int, int)` method that allows to easily grab time-activity curves. |
  | Voxel Simple data transfer object to ease the analysis of all the TACs in a given image. |
- Classes in jclustering used by jclustering.metrics

  | Class and Description |
  |  |
  | --- |
  | ImagePlusHyp This class extends `ImagePlus` in order to add a handy `ImagePlusHyp.getTAC(int, int, int)` method that allows to easily grab time-activity curves. |
  | Voxel Simple data transfer object to ease the analysis of all the TACs in a given image. |
- Classes in jclustering used by jclustering.techniques

  | Class and Description |
  |  |
  | --- |
  | Cluster Implements a cluster class. |
  | ImagePlusHyp This class extends `ImagePlus` in order to add a handy `ImagePlusHyp.getTAC(int, int, int)` method that allows to easily grab time-activity curves. |
  | Voxel Simple data transfer object to ease the analysis of all the TACs in a given image. |

- Overview
- Package
- Class
- Use
- Tree
- Deprecated
- Index
- Help

- Prev
- Next

- Frames
- No Frames

- All Classes
