# Supplementary material for: jClustering, an Open Framework for the Development of 4D Clustering Algorithms
Source: PLoS One. 2013 Aug 22;8(8):e70797. doi: 10.1371/journal.pone.0070797 (PMC3750055; doi:10.1371/journal.pone.0070797)
Supplement: File S1 — Public API for jClustering version 1.2.2. (ZIP) [file pone.0070797.s001.zip › jclustering/techniques/class-use/ClusteringTechnique.html]

Uses of Class jclustering.techniques.ClusteringTechnique


JavaScript is disabled on your browser.


- Overview
- Package
- Class
- Use
- Tree
- Deprecated
- Index
- Help

- Prev
- Next

- Frames
- No Frames

- All Classes

## Uses of Class jclustering.techniques.ClusteringTechnique

- Packages that use ClusteringTechnique

  | Package | Description |
  |  |  |
  | --- | --- |
  | jclustering |  |
  | jclustering.techniques |  |
- - ### Uses of ClusteringTechnique in jclustering

    Methods in jclustering that return ClusteringTechnique

    | Modifier and Type | Method and Description |
    |  |  |
    | --- | --- |
    | `static ClusteringTechnique` | Utils.`getClusteringTechnique(java.lang.String name, ImagePlusHyp ip, boolean skip_noisy)` Builds a new instance for a `ClusteringTechnique` object and returns it. |

    Methods in jclustering with parameters of type ClusteringTechnique

    | Modifier and Type | Method and Description |
    |  |  |
    | --- | --- |
    | `static javax.swing.JComboBox` | GUIUtils.`getMetricList(ClusteringTechnique t, ImagePlusHyp ip)` Returns a `JComboBox` of `ClusteringMetric` objects to be used inside the `ClusteringTechnique` `t`. |
  - ### Uses of ClusteringTechnique in jclustering.techniques

    Subclasses of ClusteringTechnique in jclustering.techniques

    | Modifier and Type | Class and Description |
    |  |  |
    | --- | --- |
    | `class` | `ICA` Implements an Independent Component Analysis on the image data. |
    | `class` | `KMeans` This technique implements a  k-means clustering algorithm. |
    | `class` | `LeaderFollower` Implements a leader-follower clustering method using only correlation as its main metric. |
    | `class` | `PCA` Implements a PCA clustering according to this excellent guide. |
    | `class` | `SampleTechnique` |
    | `class` | `SVD` Implements a SVD on the original image matrix. |

- Overview
- Package
- Class
- Use
- Tree
- Deprecated
- Index
- Help

- Prev
- Next

- Frames
- No Frames

- All Classes
