# Supplementary material for: jClustering, an Open Framework for the Development of 4D Clustering Algorithms
Source: PLoS One. 2013 Aug 22;8(8):e70797. doi: 10.1371/journal.pone.0070797 (PMC3750055; doi:10.1371/journal.pone.0070797)
Supplement: File S1 — Public API for jClustering version 1.2.2. (ZIP) [file pone.0070797.s001.zip › jclustering/techniques/ClusteringTechnique.html]

ClusteringTechnique


JavaScript is disabled on your browser.


- Overview
- Package
- Class
- Use
- Tree
- Deprecated
- Index
- Help

- Prev Class
- Next Class

- Frames
- No Frames

- All Classes

- Summary:
- Nested |
- Field |
- Constr |
- Method

- Detail:
- Field |
- Constr |
- Method


jclustering.techniques

## Class ClusteringTechnique

- java.lang.Object
- - jclustering.techniques.ClusteringTechnique

- All Implemented Interfaces:
  :   java.awt.event.ItemListener, java.util.EventListener

  Direct Known Subclasses:
  :   ICA, KMeans, LeaderFollower, PCA, SampleTechnique, SVD

  ---

    

  ```
  public abstract class ClusteringTechnique
  extends java.lang.Object
  implements java.awt.event.ItemListener
  ```

  This superclass should be extended by all user-implemented clustering
  techniques. If no configuration panel is needed, only the `process()`
  method must be implemented. Otherwise, the method `makeConfig()` must
  also be filled.

  Author:
  :   José María Mateos.

- - ### Constructor Summary

    Constructors

    | Constructor and Description |
    | `ClusteringTechnique()` |
  - ### Method Summary

    Methods

    | Modifier and Type | Method and Description |
    | `Cluster` | `addCluster(double[] tac)` Creates a new cluster with the `double [] tac` as the centroid, adds it to the cluster ArrayList and returns it. |
    | `void` | `addMetricsToJPanel(javax.swing.JPanel jp)` Adds a `JComboBox` element containing all the available metrics to the given panel. |
    | `void` | `addTACtoCluster(double[] tac, int x, int y, int slice, int cluster)` Provides a fast way to add a `double []` tac to a given cluster. |
    | `void` | `addTACtoCluster(Voxel v, int cluster)` Provides a fast way to add a `Voxel` to a given cluster. |
    | `void` | `compute()` This helper method is the method called from the main class. |
    | `java.lang.String[]` | `getAdditionalInfo()` Returns additional information generated by this technique in a String array. |
    | `int` | `getCloserClusterIndex(double[] tac)` Finds the cluster with a centroid as close as possible to the given TAC. |
    | `Cluster` | `getClusterAt(int index)` Provides a safe way to get the `Cluster` at the `index` position. |
    | `java.util.ArrayList<Cluster>` | `getClusters()` |
    | `javax.swing.JPanel` | `getConfig()` Builds a configuration `Panel` that will provide all the necessary interfaces for the technique configuration. |
    | `ClusteringMetric` | `getMetric()` |
    | `java.lang.String` | `getName()` |
    | `void` | `init()` Initializes the clusters ArrayList. |
    | `boolean` | `isNoise(double[] data)` Helper method. |
    | `boolean` | `isNoise(Voxel v)` Helper method. |
    | `void` | `itemStateChanged(java.awt.event.ItemEvent arg0)` |
    | `abstract void` | `process()` Performs the actual processing for this clustering technique. |
    | `void` | `setMetric(ClusteringMetric m)` Sets the current ClusteringMetric |
    | `void` | `setup(ImagePlusHyp ip)` Setup method, as the constructor will always be called empty. |
    | `void` | `skipNoisy(boolean skip_noisy)` Changes this techniques's behavior with respect to noisy voxels. |

    - ### Methods inherited from class java.lang.Object

      `equals, getClass, hashCode, notify, notifyAll, toString, wait, wait, wait`

- - ### Constructor Detail


    - #### ClusteringTechnique

      ```
      public ClusteringTechnique()
      ```
  - ### Method Detail


    - #### getName

      ```
      public java.lang.String getName()
      ```

      Returns:
      :   The name of this metric.


    - #### getConfig

      ```
      public javax.swing.JPanel getConfig()
      ```

      Builds a configuration `Panel` that will provide all the necessary
      interfaces for the technique configuration. If implemented, the technique
      class must also implement the necessary listeners.

      Developers may use the classes provided in the `GUIUtils` static
      methods.

      Returns:
      :   `null` by default, or the appropriate `Panel` if
          implemented.


    - #### process

      ```
      public abstract void process()
      ```

      Performs the actual processing for this clustering technique. This
      method fills an `ArrayList` object containing objects of the
      `Cluster` class. Each cluster contains the TACs belonging to it.
      As the `Cluster` object remembers the coordinates of every
      voxel that has been added to it, there is enough information to build
      a `ImagePlus` for representation then the processing is finished.


    - #### compute

      ```
      public void compute()
      ```

      This helper method is the method called from the main class. It
      initializes the local `Cluster` object every time a clustering
      operation is called and calls the main `process()` method.


    - #### getMetric

      ```
      public ClusteringMetric getMetric()
      ```

      Returns:
      :   The `ClusteringMetric` used by this technique, if any


    - #### setMetric

      ```
      public void setMetric(ClusteringMetric m)
      ```

      Sets the current ClusteringMetric

      Parameters:
      :   `m` - A new ClusteringMetric


    - #### getClusters

      ```
      public java.util.ArrayList<Cluster> getClusters()
      ```

      Returns:
      :   The clusters formed in this clustering technique. This method
          should be called after `process()`, which must populate
          them.


    - #### setup

      ```
      public void setup(ImagePlusHyp ip)
      ```

      Setup method, as the constructor will always be called empty. Provides a
      reference to the working image.

      Parameters:
      :   `ip` - The working image.


    - #### init

      ```
      public void init()
      ```

      Initializes the clusters ArrayList.


    - #### itemStateChanged

      ```
      public void itemStateChanged(java.awt.event.ItemEvent arg0)
      ```

      **Specified by:**
      :   `itemStateChanged` in interface `java.awt.event.ItemListener`


    - #### addMetricsToJPanel

      ```
      public void addMetricsToJPanel(javax.swing.JPanel jp)
      ```

      Adds a `JComboBox` element containing all the available metrics to the
      given panel.

      Parameters:
      :   `jp` - The panel to be modified.


    - #### skipNoisy

      ```
      public void skipNoisy(boolean skip_noisy)
      ```

      Changes this techniques's behavior with respect to noisy voxels.

      Parameters:
      :   `skip_noisy` - Boolean parameter stating whether noisy voxels should
          be discarded.


    - #### isNoise

      ```
      public boolean isNoise(double[] data)
      ```

      Helper method. Just calls `ImagePlusHyp.isNoise(double[])` method.

      Parameters:
      :   `data` - The TAC to be tested.

      Returns:
      :   true if the given TAC is noise with respect to this image.


    - #### isNoise

      ```
      public boolean isNoise(Voxel v)
      ```

      Helper method. Just calls `ImagePlusHyp.isNoise(double[])` method.

      Parameters:
      :   `v` - The voxel to be tested

      Returns:
      :   true if the given TAC is noise with respect to this image.


    - #### getClusterAt

      ```
      public Cluster getClusterAt(int index)
      ```

      Provides a safe way to get the `Cluster` at the `index`
      position.

      Parameters:
      :   `index` - The index for the cluster to be returned (1-based).

      Returns:
      :   The cluster at the given position, or a new cluster if no cluster
          exists yet at that index.


    - #### addTACtoCluster

      ```
      public void addTACtoCluster(double[] tac,
                         int x,
                         int y,
                         int slice,
                         int cluster)
      ```

      Provides a fast way to add a `double []` tac to a given cluster.

      Parameters:
      :   `tac` - The TAC data.
      :   `x` - X-coordinate for added TAC.
      :   `y` - Y-coordinate for added TAC.
      :   `slice` - Slice (1-based) for added TAC.
      :   `cluster` - The index for the cluster into which to insert the data
          (1-based).


    - #### addTACtoCluster

      ```
      public void addTACtoCluster(Voxel v,
                         int cluster)
      ```

      Provides a fast way to add a `Voxel` to a given cluster.

      Parameters:
      :   `v` - The voxel to be added.
      :   `cluster` - The index for the cluster into which to insert data
          (1-based).


    - #### getCloserClusterIndex

      ```
      public int getCloserClusterIndex(double[] tac)
      ```

      Finds the cluster with a centroid as close as possible to the given TAC.

      Parameters:
      :   `tac` - The TAC to be tested.

      Returns:
      :   The index of the cluster with the closest centroid, or -1 if none
          is found.


    - #### addCluster

      ```
      public Cluster addCluster(double[] tac)
      ```

      Creates a new cluster with the `double [] tac` as the centroid,
      adds it to the cluster ArrayList and returns it.

      Parameters:
      :   `tac` - The initial centroid for the cluster.

      Returns:
      :   The newly created cluster.


    - #### getAdditionalInfo

      ```
      public java.lang.String[] getAdditionalInfo()
      ```

      Returns additional information generated by this technique in a String
      array.

      Returns:
      :   An even-length String array with the following structure:

          - i: the suggested name for the file used to store this info.
          - i+1: the actual contents of the additional information.where `i` starts at 0. All the file names occupy the
          even-numbered indexes of the array. File names cannot be
          `null`.


- Overview
- Package
- Class
- Use
- Tree
- Deprecated
- Index
- Help

- Prev Class
- Next Class

- Frames
- No Frames

- All Classes

- Summary:
- Nested |
- Field |
- Constr |
- Method

- Detail:
- Field |
- Constr |
- Method
