# Supplementary material for: jClustering, an Open Framework for the Development of 4D Clustering Algorithms
Source: PLoS One. 2013 Aug 22;8(8):e70797. doi: 10.1371/journal.pone.0070797 (PMC3750055; doi:10.1371/journal.pone.0070797)
Supplement: File S1 — Public API for jClustering version 1.2.2. (ZIP) [file pone.0070797.s001.zip › jclustering/techniques/LeaderFollower.html]

LeaderFollower


JavaScript is disabled on your browser.


- Overview
- Package
- Class
- Use
- Tree
- Deprecated
- Index
- Help

- Prev Class
- Next Class

- Frames
- No Frames

- All Classes

- Summary:
- Nested |
- Field |
- Constr |
- Method

- Detail:
- Field |
- Constr |
- Method


jclustering.techniques

## Class LeaderFollower

- java.lang.Object
- - jclustering.techniques.ClusteringTechnique
  - - jclustering.techniques.LeaderFollower

- All Implemented Interfaces:
  :   java.awt.event.FocusListener, java.awt.event.ItemListener, java.util.EventListener

  ---

    

  ```
  public class LeaderFollower
  extends ClusteringTechnique
  implements java.awt.event.FocusListener
  ```

  Implements a leader-follower clustering method using only correlation
  as its main metric. Also uses the mean peak value of all the TACs inside
  a Cluster to decide whether a certain TAC belongs or not to a voxel
  with similar dynamic shape.

  Leader-follower works in an inverse way as K-means does. The number of
  clusters is unknown, and a threshold is set so that clusters are formed
  with TACs with a distance (correlation, in this case) that evaluates
  above said threshold. An optional increment value can be set so that
  every time a voxel is added to a cluster this becomes more restrictive.

  Also, peak amplitude is taken into account when adding new TACs to an
  existing cluster.

  Author:
  :   José María Mateos.

- - ### Constructor Summary

    Constructors

    | Constructor and Description |
    | `LeaderFollower()` |
  - ### Method Summary

    Methods

    | Modifier and Type | Method and Description |
    | `void` | `focusGained(java.awt.event.FocusEvent arg0)` |
    | `void` | `focusLost(java.awt.event.FocusEvent arg0)` |
    | `void` | `itemStateChanged(java.awt.event.ItemEvent arg0)` |
    | `javax.swing.JPanel` | `makeConfig()` This function is called only once and returns the configuration panel that will be called by `#getConfig()` on each successive call. |
    | `void` | `process()` Performs the actual processing for this clustering technique. |

    - ### Methods inherited from class jclustering.techniques.ClusteringTechnique

      `addCluster, addMetricsToJPanel, addTACtoCluster, addTACtoCluster, compute, getAdditionalInfo, getCloserClusterIndex, getClusterAt, getClusters, getConfig, getMetric, getName, init, isNoise, isNoise, setMetric, setup, skipNoisy`
    - ### Methods inherited from class java.lang.Object

      `equals, getClass, hashCode, notify, notifyAll, toString, wait, wait, wait`

- - ### Constructor Detail


    - #### LeaderFollower

      ```
      public LeaderFollower()
      ```
  - ### Method Detail


    - #### process

      ```
      public void process()
      ```

      **Description copied from class: `ClusteringTechnique`**

      Performs the actual processing for this clustering technique. This
      method fills an `ArrayList` object containing objects of the
      `Cluster` class. Each cluster contains the TACs belonging to it.
      As the `Cluster` object remembers the coordinates of every
      voxel that has been added to it, there is enough information to build
      a `ImagePlus` for representation then the processing is finished.

      **Specified by:**
      :   `process` in class `ClusteringTechnique`


    - #### makeConfig

      ```
      public javax.swing.JPanel makeConfig()
      ```

      **Description copied from class: `ClusteringTechnique`**

      This function is called only once and returns the configuration panel
      that will be called by `#getConfig()` on each successive call.
      Needs to be overridden by the extending classes.

      Returns:
      :   The configuration panel returned by `#getConfig()`.


    - #### focusGained

      ```
      public void focusGained(java.awt.event.FocusEvent arg0)
      ```

      **Specified by:**
      :   `focusGained` in interface `java.awt.event.FocusListener`


    - #### focusLost

      ```
      public void focusLost(java.awt.event.FocusEvent arg0)
      ```

      **Specified by:**
      :   `focusLost` in interface `java.awt.event.FocusListener`


    - #### itemStateChanged

      ```
      public void itemStateChanged(java.awt.event.ItemEvent arg0)
      ```

      **Specified by:**
      :   `itemStateChanged` in interface `java.awt.event.ItemListener`

      **Overrides:**
      :   `itemStateChanged` in class `ClusteringTechnique`


- Overview
- Package
- Class
- Use
- Tree
- Deprecated
- Index
- Help

- Prev Class
- Next Class

- Frames
- No Frames

- All Classes

- Summary:
- Nested |
- Field |
- Constr |
- Method

- Detail:
- Field |
- Constr |
- Method
