# Supplementary material for: jClustering, an Open Framework for the Development of 4D Clustering Algorithms
Source: PLoS One. 2013 Aug 22;8(8):e70797. doi: 10.1371/journal.pone.0070797 (PMC3750055; doi:10.1371/journal.pone.0070797)
Supplement: File S1 — Public API for jClustering version 1.2.2. (ZIP) [file pone.0070797.s001.zip › jclustering/techniques/package-summary.html]

jclustering.techniques


JavaScript is disabled on your browser.


- Overview
- Package
- Class
- Use
- Tree
- Deprecated
- Index
- Help

- Prev Package
- Next Package

- Frames
- No Frames

- All Classes

# Package jclustering.techniques

- Class Summary

  | Class | Description |
  |  |  |
  | --- | --- |
  | ClusteringTechnique | This superclass should be extended by all user-implemented clustering techniques. |
  | ICA | Implements an Independent Component Analysis on the image data. |
  | KMeans | This technique implements a  k-means clustering algorithm. |
  | LeaderFollower | Implements a leader-follower clustering method using only correlation as its main metric. |
  | PCA | Implements a PCA clustering according to this excellent guide. |
  | SampleTechnique |  |
  | SVD | Implements a SVD on the original image matrix. |

- Overview
- Package
- Class
- Use
- Tree
- Deprecated
- Index
- Help

- Prev Package
- Next Package

- Frames
- No Frames

- All Classes
