# Supplementary material for: jClustering, an Open Framework for the Development of 4D Clustering Algorithms
Source: PLoS One. 2013 Aug 22;8(8):e70797. doi: 10.1371/journal.pone.0070797 (PMC3750055; doi:10.1371/journal.pone.0070797)
Supplement: File S1 — Public API for jClustering version 1.2.2. (ZIP) [file pone.0070797.s001.zip › jclustering/TimeVectorReader.html]

TimeVectorReader


JavaScript is disabled on your browser.


- Overview
- Package
- Class
- Use
- Tree
- Deprecated
- Index
- Help

- Prev Class
- Next Class

- Frames
- No Frames

- All Classes

- Summary:
- Nested |
- Field |
- Constr |
- Method

- Detail:
- Field |
- Constr |
- Method


jclustering

## Class TimeVectorReader

- java.lang.Object
- - jclustering.TimeVectorReader

- ---

    

  ```
  public class TimeVectorReader
  extends java.lang.Object
  ```

  This class reads data from a text file with two columns separated by a
  space: each row contains the starting time (first column) and ending time
  (second column). Example:

  ```
   1.0 2.0
   2.0 3.0
   3.0 4.0
   ...
  ```

  This file needs to have as many rows as frames, or the time data will
  not be used when saving data to file.

  This class can also read PMOD's .acqtimes files.

  Author:
  :   José María Mateos.

- - ### Constructor Summary

    Constructors

    | Constructor and Description |
    | `TimeVectorReader(java.lang.String file_path)` Builds a new TimeVectorReader object. |
  - ### Method Summary

    Methods

    | Modifier and Type | Method and Description |
    | `double[][]` | `getTimeVector()` Reads the data provided in the constructor and returns the appropriate time vector array. |

    - ### Methods inherited from class java.lang.Object

      `equals, getClass, hashCode, notify, notifyAll, toString, wait, wait, wait`

- - ### Constructor Detail


    - #### TimeVectorReader

      ```
      public TimeVectorReader(java.lang.String file_path)
      ```

      Builds a new TimeVectorReader object.

      Parameters:
      :   `file_path` - The path for the file that includes the time vector
          data.
  - ### Method Detail


    - #### getTimeVector

      ```
      public double[][] getTimeVector()
      ```

      Reads the data provided in the constructor and returns the appropriate
      time vector array.

      Returns:
      :   A time vector array, or null if there is some problem or the
          path used in the constructor was null.


- Overview
- Package
- Class
- Use
- Tree
- Deprecated
- Index
- Help

- Prev Class
- Next Class

- Frames
- No Frames

- All Classes

- Summary:
- Nested |
- Field |
- Constr |
- Method

- Detail:
- Field |
- Constr |
- Method
