# Supplementary material for: jClustering, an Open Framework for the Development of 4D Clustering Algorithms
Source: PLoS One. 2013 Aug 22;8(8):e70797. doi: 10.1371/journal.pone.0070797 (PMC3750055; doi:10.1371/journal.pone.0070797)
Supplement: File S1 — Public API for jClustering version 1.2.2. (ZIP) [file pone.0070797.s001.zip › jclustering/Utils.html]

Utils


JavaScript is disabled on your browser.


- Overview
- Package
- Class
- Use
- Tree
- Deprecated
- Index
- Help

- Prev Class
- Next Class

- Frames
- No Frames

- All Classes

- Summary:
- Nested |
- Field |
- Constr |
- Method

- Detail:
- Field |
- Constr |
- Method


jclustering

## Class Utils

- java.lang.Object
- - jclustering.Utils

- ---

    

  ```
  public class Utils
  extends java.lang.Object
  ```

  Auxiliary class with misc static methods

  Author:
  :   José María Mateos

- - ### Constructor Summary

    Constructors

    | Constructor and Description |
    | `Utils()` |
  - ### Method Summary

    Methods

    | Modifier and Type | Method and Description |
    | `static ij.ImagePlus` | `createImagePlus(int x, int y, int z, int nFrames)` Creates an empty ImagePlus with the given dimensions. |
    | `static ij.ImagePlus` | `expand(ij.ImagePlus ip, int cluster_number)` Transforms a static `ImagePlus` object into a dynamic one, with the correct structure for a cluster result. |
    | `static java.util.ArrayList<java.lang.String>` | `getAllMetrics()` Finds all the classes that extend the ClusteringMetric superclass and returns a list of their names. |
    | `static java.util.ArrayList<java.lang.String>` | `getAllTechniques()` Finds all the classes that extend the ClusteringTechnique superclass and returns a list of their names. |
    | `static ClusteringMetric` | `getClusteringMetric(java.lang.String name, ImagePlusHyp ip)` Builds a new instance for a `ClusteringMetric` object and returns it. |
    | `static ClusteringTechnique` | `getClusteringTechnique(java.lang.String name, ImagePlusHyp ip, boolean skip_noisy)` Builds a new instance for a `ClusteringTechnique` object and returns it. |
    | `static <T> java.lang.String` | `getName(T c)` Returns the name of a given class, without the package name |
    | `static ij.ImagePlus` | `RealMatrix2IJ(org.apache.commons.math3.linear.RealMatrix rm, int[] dim, ImagePlusHyp ip, boolean skip_noisy, java.lang.String name)` Transforms a `RealMatrix` object into a ImageJ image. |
    | `static void` | `setVoxel(ij.ImageStack is, int x, int y, int slice, double value)` Provides a handy method for setting voxel values using slices as z-indexes (which are 0-based). |

    - ### Methods inherited from class java.lang.Object

      `equals, getClass, hashCode, notify, notifyAll, toString, wait, wait, wait`

- - ### Constructor Detail


    - #### Utils

      ```
      public Utils()
      ```
  - ### Method Detail


    - #### getAllTechniques

      ```
      public static java.util.ArrayList<java.lang.String> getAllTechniques()
      ```

      Finds all the classes that extend the ClusteringTechnique superclass and
      returns a list of their names.

      Returns:
      :   An ArrayList containing all the class names.


    - #### getAllMetrics

      ```
      public static java.util.ArrayList<java.lang.String> getAllMetrics()
      ```

      Finds all the classes that extend the ClusteringMetric superclass and
      returns a list of their names.

      Returns:
      :   An ArrayList containing all the class names.


    - #### getClusteringTechnique

      ```
      public static ClusteringTechnique getClusteringTechnique(java.lang.String name,
                                               ImagePlusHyp ip,
                                               boolean skip_noisy)
      ```

      Builds a new instance for a `ClusteringTechnique` object and
      returns it.

      Parameters:
      :   `name` - The name of the ClusteringTechnique to build.
      :   `ip` - A reference to the working image.
      :   `skip_noisy` - True if noisy voxels are to be skipped.

      Returns:
      :   A new instance of the said ClusteringTechnique.


    - #### getClusteringMetric

      ```
      public static ClusteringMetric getClusteringMetric(java.lang.String name,
                                         ImagePlusHyp ip)
      ```

      Builds a new instance for a `ClusteringMetric` object and returns
      it.

      Parameters:
      :   `name` - The name of the ClusteringMetric to build.
      :   `ip` - A reference to the working image.

      Returns:
      :   A new instance of the said ClusteringMetric.


    - #### getName

      ```
      public static <T> java.lang.String getName(T c)
      ```

      Returns the name of a given class, without the package name

      Parameters:
      :   `c` - The class

      Returns:
      :   The name of the class


    - #### createImagePlus

      ```
      public static ij.ImagePlus createImagePlus(int x,
                                 int y,
                                 int z,
                                 int nFrames)
      ```

      Creates an empty ImagePlus with the given dimensions. The resulting image
      will have a bit depth of 8 bits, which should be enough to store all the
      clusters (maximum clusters = 255).

      Parameters:
      :   `x` - Width, in pixels.
      :   `y` - Height, in pixels.
      :   `z` - Number of slices.
      :   `nFrames` - Number of frames.

      Returns:
      :   An empty ImagePlus object.


    - #### expand

      ```
      public static ij.ImagePlus expand(ij.ImagePlus ip,
                        int cluster_number)
      ```

      Transforms a static `ImagePlus` object into a dynamic one, with the
      correct structure for a cluster result.

      Parameters:
      :   `ip` - The static `ImagePlus` obtained from the
          `ClusteringTechnique.process()` method.
      :   `cluster_number` - The number of clusters.

      Returns:
      :   A clustering result, dynamic `ImagePlus`.


    - #### setVoxel

      ```
      public static void setVoxel(ij.ImageStack is,
                  int x,
                  int y,
                  int slice,
                  double value)
      ```

      Provides a handy method for setting voxel values using slices as
      z-indexes (which are 0-based).

      Parameters:
      :   `is` - The `ImageStack` to be modified
      :   `x` - x-coordinate
      :   `y` - y-coordinate
      :   `slice` - Slice number (1-based).
      :   `value` - The value to be set.


    - #### RealMatrix2IJ

      ```
      public static ij.ImagePlus RealMatrix2IJ(org.apache.commons.math3.linear.RealMatrix rm,
                               int[] dim,
                               ImagePlusHyp ip,
                               boolean skip_noisy,
                               java.lang.String name)
      ```

      Transforms a `RealMatrix` object into a ImageJ image.

      Parameters:
      :   `rm` - The RealMatrix to be converted.
      :   `dim` - The desired dimensions for the final image
      :   `ip` - A reference to the image object that generated this operation
      :   `skip_noisy` - Should noisy voxels be skipped?
      :   `name` - The name for the new image.

      Returns:
      :   The newly generated `ImagePlus` object.


- Overview
- Package
- Class
- Use
- Tree
- Deprecated
- Index
- Help

- Prev Class
- Next Class

- Frames
- No Frames

- All Classes

- Summary:
- Nested |
- Field |
- Constr |
- Method

- Detail:
- Field |
- Constr |
- Method
