# Supplementary material for: jClustering, an Open Framework for the Development of 4D Clustering Algorithms
Source: PLoS One. 2013 Aug 22;8(8):e70797. doi: 10.1371/journal.pone.0070797 (PMC3750055; doi:10.1371/journal.pone.0070797)
Supplement: File S1 — Public API for jClustering version 1.2.2. (ZIP) [file pone.0070797.s001.zip › jclustering/Voxel.html]

Voxel


JavaScript is disabled on your browser.


- Overview
- Package
- Class
- Use
- Tree
- Deprecated
- Index
- Help

- Prev Class
- Next Class

- Frames
- No Frames

- All Classes

- Summary:
- Nested |
- Field |
- Constr |
- Method

- Detail:
- Field |
- Constr |
- Method


jclustering

## Class Voxel

- java.lang.Object
- - jclustering.Voxel

- ---

    

  ```
  public class Voxel
  extends java.lang.Object
  ```

  Simple data transfer object to ease the analysis of all the TACs in a
  given image. Objects returned by the `ImagePlusHypIterator` class
  are of this type.

  Author:
  :   José María Mateos.

- - ### Field Summary

    Fields

    | Modifier and Type | Field and Description |
    | `int` | `slice` Slice (1-based) for the voxel. |
    | `double[]` | `tac` Time-activity curve. |
    | `int` | `x` X-coordinate for the voxel. |
    | `int` | `y` Y-coordinate for the voxel. |
  - ### Constructor Summary

    Constructors

    | Constructor and Description |
    | `Voxel(int x, int y, int slice, double[] tac)` Main constructor for this data transfer object |
  - ### Method Summary

    - ### Methods inherited from class java.lang.Object

      `equals, getClass, hashCode, notify, notifyAll, toString, wait, wait, wait`

- - ### Field Detail


    - #### x

      ```
      public final int x
      ```

      X-coordinate for the voxel.


    - #### y

      ```
      public final int y
      ```

      Y-coordinate for the voxel.


    - #### slice

      ```
      public final int slice
      ```

      Slice (1-based) for the voxel.


    - #### tac

      ```
      public final double[] tac
      ```

      Time-activity curve.
  - ### Constructor Detail


    - #### Voxel

      ```
      public Voxel(int x,
           int y,
           int slice,
           double[] tac)
      ```

      Main constructor for this data transfer object

      Parameters:
      :   `x` - X-coordinate.
      :   `y` - Y-coordinate.
      :   `slice` - Slice number.
      :   `tac` - Time-activity curve.


- Overview
- Package
- Class
- Use
- Tree
- Deprecated
- Index
- Help

- Prev Class
- Next Class

- Frames
- No Frames

- All Classes

- Summary:
- Nested |
- Field |
- Constr |
- Method

- Detail:
- Field |
- Constr |
- Method
