# Supplementary material for: jClustering, an Open Framework for the Development of 4D Clustering Algorithms
Source: PLoS One. 2013 Aug 22;8(8):e70797. doi: 10.1371/journal.pone.0070797 (PMC3750055; doi:10.1371/journal.pone.0070797)
Supplement: File S1 — Public API for jClustering version 1.2.2. (ZIP) [file pone.0070797.s001.zip › overview-summary.html]

Overview


JavaScript is disabled on your browser.


- Overview
- Package
- Class
- Use
- Tree
- Deprecated
- Index
- Help

- Prev
- Next

- Frames
- No Frames

- All Classes

# jClustering

jClustering is a clustering framework developed for ImageJ.

See: Description

Packages

| Package | Description |
|  |  |
| --- | --- |
| jclustering |  |
| jclustering.metrics |  |
| jclustering.techniques |  |

jClustering is a clustering framework developed for ImageJ. It aims at
providing an easy to extend platform in which to develop new clustering
algorithms. You can find a comprehensive manual for this plugin on
its main page at
github, including documentation for developers.

- Overview
- Package
- Class
- Use
- Tree
- Deprecated
- Index
- Help

- Prev
- Next

- Frames
- No Frames

- All Classes
