# Supplementary material for: jClustering, an Open Framework for the Development of 4D Clustering Algorithms
Source: PLoS One. 2013 Aug 22;8(8):e70797. doi: 10.1371/journal.pone.0070797 (PMC3750055; doi:10.1371/journal.pone.0070797)
Supplement: File S1 — Public API for jClustering version 1.2.2. (ZIP) [file pone.0070797.s001.zip › overview-tree.html]

Class Hierarchy


JavaScript is disabled on your browser.


- Overview
- Package
- Class
- Use
- Tree
- Deprecated
- Index
- Help

- Prev
- Next

- Frames
- No Frames

- All Classes

# Hierarchy For All Packages

Package Hierarchies:

- jclustering,
- jclustering.metrics,
- jclustering.techniques

## Class Hierarchy

- java.lang.Object
  - jclustering.Cluster
  - jclustering.metrics.ClusteringMetric (implements java.awt.event.ActionListener, java.awt.event.ItemListener)
    - jclustering.metrics.Correlation
    - jclustering.metrics.Mahalanobis
    - jclustering.metrics.PNorm (implements java.awt.event.FocusListener)
    - jclustering.metrics.RMSD
  - jclustering.techniques.ClusteringTechnique (implements java.awt.event.ItemListener)
    - jclustering.techniques.ICA (implements java.awt.event.FocusListener)
    - jclustering.techniques.KMeans (implements java.awt.event.FocusListener)
    - jclustering.techniques.LeaderFollower (implements java.awt.event.FocusListener)
    - jclustering.techniques.PCA
    - jclustering.techniques.SampleTechnique
    - jclustering.techniques.SVD
  - jclustering.Constants
  - jclustering.FileSaver
  - jclustering.GUIUtils
  - ij.ImagePlus (implements java.lang.Cloneable, java.awt.image.ImageObserver, ij.measure.Measurements)
    - jclustering.ImagePlusHyp (implements java.lang.Iterable<T>)
  - jclustering.ImagePlusHypIterator (implements java.util.Iterator<E>)
  - jclustering.JClustering\_ (implements java.awt.event.ActionListener, java.awt.event.ComponentListener, java.awt.event.ItemListener, ij.plugin.filter.PlugInFilter)
  - jclustering.MathUtils
  - jclustering.TimeVectorReader
  - jclustering.Utils
  - jclustering.Voxel

- Overview
- Package
- Class
- Use
- Tree
- Deprecated
- Index
- Help

- Prev
- Next

- Frames
- No Frames

- All Classes
