# Supplementary material for: jClustering, an Open Framework for the Development of 4D Clustering Algorithms
Source: PLoS One. 2013 Aug 22;8(8):e70797. doi: 10.1371/journal.pone.0070797 (PMC3750055; doi:10.1371/journal.pone.0070797)
Supplement: File S2 — Example of simple ClusteringTechnique class. (DOCX) [file pone.0070797.s002.docx]

# Supporting Information 2: Example of clustering

The code below generates a *ClusteringTechnique* that groups voxels taking into account only their time to peak value:

package jclustering.techniques;

import jclustering.Voxel;

public class SampleTechnique extends ClusteringTechnique {

public void process() {

for (Voxel v : ip) {

int n = _getMaxIndex(v.tac) + 1;

addTACtoCluster(v, n); // Add TAC v to Cluster n

}

}

// Helper method

private int _getMaxIndex(double[] d) {

int res = 0;

double aux = d[0];

for (int i = 0; i < d.length; i++) {

if (d[i] > aux) {

res = i;

aux = d[i];

}

}

return res;

}

}
